# Language‐Invariant Strategies of Navigating Transitions in Joint Activities: Forms and Functions of Coordination Markers

**DOI:** 10.1111/cogs.70133

**Published:** 2025-11-26

**Authors:** Natalia Morozova, Sabine Stoll, Adrian Bangerter

**Affiliations:** ^1^ Institute for the Interdisciplinary Study of Language Evolution (ISLE Institute) University of Zurich; ^2^ Institute of Work and Organizational Psychology (IPTO) University of Neuchâtel

**Keywords:** Joint action, Coordination, Human cooperation, Convergent evolution, Coordination markers

## Abstract

Goal‐directed tasks unfold in hierarchies of larger and smaller sub‐tasks, and pursuing them jointly implies that participants must agree on whether they are continuing an ongoing sub‐task (horizontal transition) or switching to the next sub‐task (vertical transition). Previous research indicates that humans employ short and efficient coordination markers as procedural conventions to distinguish horizontal (e.g., in English, with *yeah* and *uh‐huh*) and vertical transitions (with *okay*, *all right*). However, it remains unclear (1) whether such words serve as potentially universal coordination devices and (2) which properties make some markers more suitable for horizontal versus vertical transition contexts. We hypothesized that horizontal transitions in ongoing sub‐tasks are associated with higher dual‐tasking interference between verbal coordination and the nonlinguistic task, therefore, constraining the lexicality of coordination markers. In our experimental study, we assessed how speakers of three typologically diverse languages (Swiss French, Vietnamese, and Shipibo‐Konibo; *N* = 232) used coordination markers to navigate a joint LEGO‐building task. We found that in each language, coordination markers comprise a system of transition‐specific conventions and that participants strategically deployed markers with minimal lexical and acoustic forms (*uh‐huh, mm*) and repetitions in horizontal transitions, while more lexicalized markers (e.g., *okay*) in vertical transitions. Our findings suggest that (1) coordination markers are potentially universal linguistic devices for navigating joint activities and (2) the forms of coordination markers might be shaped by the constraints of their primary interaction context (here, horizontal and vertical transitions). Our study provides new evidence of how interactional settings might selectively shape language use through the forces of convergent language evolution.

## 1 Introduction

Cooperative, or joint, activities constitute the primary communicative environment, or the ecological niche, in which human language emerged and subsequently evolved (Croft, 2000; Levinson, 2006; Roberts & Levinson, 2017; Schegloff, 2020; Tomasello, 2010). As language serves to support and regulate joint activities (Clark, 1996; Knutsen, Bangerter, & Mayor, 2019; Mills, 2014), the generic (and potentially universal; Levinson, 2006) infrastructure of joint activities exerts functional pressures on how humans use language to coordinate them (Croft, 2000; Dingemanse, Torreira, & Enfield, 2013; Roberts & Levinson, 2017). In other words, universally recurring coordination requirements emerging in joint activities presumably lead speakers of different languages to develop similar linguistic solutions, a case of convergent evolution of linguistic items (Dingemanse et al., 2015). For instance, to establish and dissolve the commitment to pursue a joint goal (Bangerter, Genty, Heesen, Rossano, & Zuberbühler, 2022), humans in many cultures use greeting conventions (Duranti, 1997) to open joint interactions and leave‐taking rituals (Albert & Kessler, 1976; Broth & Mondada, 2013) to close them. Verbal contributions in social interactions are universally organized into adjacency‐pair sequences (Kendrick et al., 2020; Schegloff, 2007) and are produced with minimal gap and overlap between speech turns (Sacks, Schegloff, & Jefferson, 1974; Stivers et al., 2009). Furthermore, some linguistic conventions regulating joint actions exhibit striking convergence of lexical forms across many unrelated languages, for example, repair particle *huh?* (Dingemanse et al., 2013) and turn‐regulating markers *uh‐huh, mm* that indicate understanding and continuation of the conversation (Dingemanse, Liesenfeld, & Woensdregt, 2022).

In this paper, we explore how yet another universal coordination constraint—signaling transitions through the sub‐components of an activity (Bangerter & Clark, 2003)—that might have shaped language use through the forces of convergent evolution. As any joint activity unfolds, participants coordinate the progress through their activity by distinguishing the continuation of its ongoing sub‐components (i.e., horizontal transitions) from openings and closings of sub‐components (i.e., vertical transitions; Bangerter & Clark, 2003). If human language evolved to facilitate the coordination of joint activities (Clark, 1996), we propose that such a recurrent coordination problem might have led to the convergence of linguistic conventions (here, *coordination markers*) used to signal horizontal and vertical transitions, even in unrelated languages.

We proceed as follows: first, we review how humans abstract goal‐directed activities into hierarchies of larger and smaller sub‐components and describe two generic types of transitions through this hierarchy, that is, horizontal transitions within and vertical transitions between sub‐components of an activity (Section 1.1). Further, we discuss which coordination markers (words like *uh‐huh*, *yeah*, or *okay* in English) humans use to signal horizontal and vertical transitions (Section 1.2) and how the differences in functional pressures exerted by horizontal versus vertical transition contexts might shape the choice of coordination markers (Section 1.3). We hypothesize (Section 1.4) that (a) coordination markers specialized for horizontal (e.g., *uh‐huh* in English) and vertical (e.g., *okay* in English) transitions could be found in many languages and that (b) their linguistic forms might exhibit some language‐invariant properties, presumably shaped by similar functional pressures of their interaction contexts. We tested (Section 2) these hypotheses in an experimental study, which examined whether coordination markers produced during a collaborative LEGO‐building task are specialized for marking horizontal or vertical transition contexts. Participants were speakers of three typologically diverse languages: Vietnamese, Swiss French, and Shipibo‐Konibo.

Our findings (Section 3) suggest that coordination markers may represent a universal class of linguistic conventions. Although they are not exclusive devices for navigating the hierarchy of joint activities, the use of coordination markers in each target language reflects the distinction between two different transitions, and their transition‐specialized linguistic forms exhibit some language‐invariant properties, even in maximally diverse languages. This study enhances our understanding of the role of cooperation in human language evolution and illustrates how external functional pressures of specific interaction contexts can shape language use. Such findings might find application in automatic recognition of speech acts and set the ground for further exploration of language‐invariant outcomes of coordination markers, for example, in the domains of phonetics or language development.

### 1.1 Joint activities as hierarchically organized tasks and sub‐tasks

Humans deploy event cognition to plan and process goal‐directed actions (Zacks & Tversky, 2001). A large body of research suggests that goal‐directed activities are abstracted into an ordered sequence of distinct events to facilitate action planning (e.g., Correa, Ho, Callaway, Daw, & Griffiths, 2023; Solway et al., 2014; Tomov, Yagati, Kumar, Yang, & Gershman, 2020) and to aid in perceiving and interpreting the actions of others (Hard, Recchia, & Tversky, 2011; Newtson & Engquist, 1976; Zacks et al., 2001). These abstractions represent clusters of tasks, sub‐tasks, and sub‐subtasks. For example, the task of *building a three‐block LEGO model* could unfold in a sequence of sub‐tasks *block one*, *block two*, *block three*. Each of these sub‐tasks, in turn, would include sub‐subtasks of individual construction steps, for example, *finding the block, placing it, adjusting its position*.


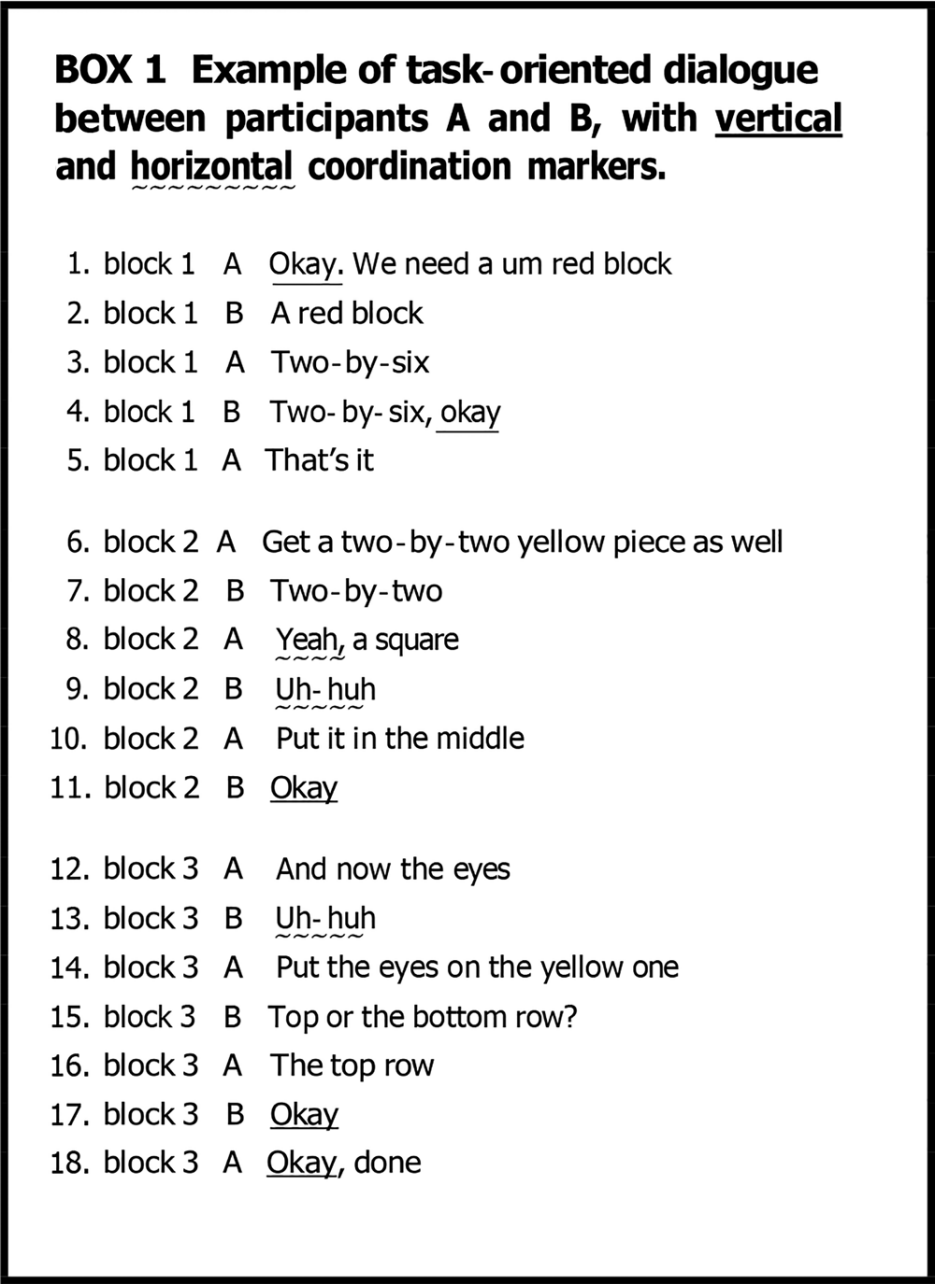


In joint activities, participants must coordinate their individual actions in time and space to achieve a shared goal (Sebanz, Bekkering, & Knoblich, 2006). Navigating joint activities, therefore, implies that the interacting parties must coordinate when and how to transition from one part of their activity to the next. In the joint task of *building a LEGO model together*, participants might transition through its sub‐tasks **horizontally**, that is, by continuing the construction steps within one sub‐task (*identifying* — *finding* — *placing a block* — *adjusting its placement*), or **vertically** between block sub‐tasks, for example, by finishing *block one* and moving to *block two* (Bangerter & Clark, 2003). Participants thus need to agree whether they are horizontally continuing an ongoing sub‐task or vertically finishing it and moving to the next sub‐task. Box 1 illustrates how participants establish and continuously update these agreements. Each contribution to the joint task, for example, placement instruction (10) for *block two*, could be grounded (Clark & Schaefer, 1987, 1989) horizontally by inviting a new contribution to the sub‐task *block two*, for example, *Uh‐huh, nothing is sticking out, right?*, or vertically by agreeing to move to *block three*, for example, *Okay, what's next?*

Participants may explicitly ratify each other's contributions. For example, instead of (9), B might say *I've found a yellow square, please continue*, or instead of (11) *I've put it in the middle, now we can move to the next block*. However, this would be effortful as participants would need to ground each contribution to continuously update their mutual understanding of progress in the joint activity. Alternatively, they could ground these transitions in a more implicit way, via using *coordination markers*.

### 1.2 Coordination markers as procedural conventions for navigating joint activities

To navigate the procedural order of individual contributions, participants in joint tasks rely on well‐established and recognized conventions (Clark, 1996) or collaboratively select novel conventions as an emergent solution to procedural coordination problems (Mills, 2011). There is evidence that (1) words like *uh‐huh*, *yeah*, *right*, *all right*, and *okay* function as conventionalized coordination markers that signal transitions in the course of a joint activity and that (2) each of these markers is specialized either for horizontal or vertical transition contexts (Bangerter & Clark, 2003). The category of coordination markers encompasses conversational tokens that are produced by both speakers and listeners and signal (i) understanding (*uh‐huh*, *yeah*), (ii) acceptance (*okay*, *all right*), or (iii) assessment (*perfect*, *cool*) to ground and ratify contributions to a joint activity. These words have been studied in various ways, including as continuers (Schegloff, 1982), backchannel responses (Yngve, 1970), discourse markers (Schiffrin, 1987), and acknowledgment tokens (Jefferson, 1984). They are frequently used in task‐oriented dialogues and serve to maintain alignment between participants (Bangerter & Clark, 2003; Dideriksen et al., 2023; Fusaroli et al., 2017; Knutsen et al., 2019; Spaniol, Wehrle, Janz, Vogeley, & Grice, 2024). Although they overlap in meaning and function, participants do not always use them interchangeably. For example, in English, by responding with *okay* and *yeah*, listeners often imply that they are ready to take the floor, while *uh‐huh* is mostly used as a turn‐foregoing response, encouraging the current speaker to continue (Drummond & Hopper, 1993; Jurafsky, Shriberg, Fox, & Curl, 1998).

By subsuming these words into the category of coordination markers, it becomes apparent that they are specialized for specific transition contexts (Bangerter & Clark, 2003). English speakers preferentially use *uh‐huh*, *mm‐hm*, and *yeah* in horizontal transitions. *Okay* and *all right*, in contrast, are mostly found in vertical transition contexts. Hence, *uh‐huh*, *mm‐hm*, and *yeah* function as conventionalized **horizontal markers** and *okay* and *all right* as **vertical markers**, as illustrated in Box 1. Vertical use of *okay* has been observed in a number of other languages, for example, Swiss French (Bangerter, Knutsen, Germanier, Col, & Brosy, 2024) and Swiss German (Bangerter & Clark, 2003), Brazilian Portuguese, Chinese Mandarin, Danish, Estonian, Italian, Japanese, Korean, Polish, and Swedish (for a review, Mondada & Sorjonen, 2021). There are many conversational contexts where vertical *okay* was documented cross‐linguistically: in openings and closings of conversations, during the change of conversational topics, and in closing segments of topic digressions.

Despite the lack of cross‐linguistic studies documenting the repertoires of horizontal and vertical markers, it appears that the distribution of coordination marker forms between two transition types might not be arbitrary. As discussed above, speakers of many languages prefer using *okay* in vertical contexts, and words like *uh‐huh, yeah, mm* frequently appear in horizontal conversational contexts as markers of continuation (Dingemanse et al., 2022). We further discuss whether horizontal and vertical transitions might pose different selective pressures on the planning and processing of coordination markers and how certain formal properties of these markers could make them more suitable candidates for appearing in specific transitions.

### 1.3 Dual‐tasking constraints and the lexicality of coordination markers

In task‐oriented conversation, participants must simultaneously allocate attention between the task steps and verbal coordination, which entails engaging in so‐called dual‐tasking. In this context, dual‐tasking refers to the simultaneous execution of two (linguistic and nonlinguistic) tasks, often causing one task to interfere with the other (Pernon, Fournet, Fougeron, & Laganaro, 2019). Accordingly, participants' performance in a concurrent nonlinguistic task may decline as they converse (Sjerps & Meyer, 2015), with greater impact on speech planning than on listening (Boiteau, Malone, Peters, & Almor, 2014).

Since horizontal transitions fall within ongoing sub‐tasks of a joint activity, the production of horizontal coordination markers must be more constrained by dual‐tasking pressures than the production of vertical markers. In order to mitigate the dual‐tasking processing load, participants might prioritize the production of coordination markers that are (1) easier to plan and produce under increased processing constraints and (2) allow to maintain the focus of attention on the task at hand. From previous research, we know of several strategies that allow interlocutors to achieve these goals. First, there is indirect evidence that markers with minimal lexical and acoustic forms (*mm‐hm, uh‐huh, mm*) might require less effort to plan and produce. Unlike content‐bearing words, these minimal markers represent a closed class of conventions that serve to express understanding rather than introducing new content into the conversation (Knudsen, Creemers, & Meyer, 2020). Such generic markers of understanding and continuation are also more likely to be used under the constraints of additional mental load (Bavelas, Coates, & Johnson, 2000). For current floor holders, minimal markers should be easier to process as they are often produced in overlap as unobtrusive, turn‐foregoing signals (Dingemanse et al., 2022; Jefferson, 1984; Jurafsky et al., 1998; McCarthy, 2003; Schegloff, 1982), and thus do not prompt the shift of speaker's attention from the current topic (or sub‐task). Similarly, repetitions function as turn‐foregoing continuers, serving to display understanding or registering the information (Couper‐Kuhlen & Selting, 2017; Svennevig, 2004; Zellers, 2021). Repetitions may also reduce working memory load (Clark & Schaefer, 1989) or alleviate speech planning through lexical priming (Bartolozzi, Jongman, & Meyer, 2021), potentially easing dual‐tasking constraints. Alternatively, participants might use the visual modality to achieve grounding more efficiently, for instance, by using gaze, nodding, gesturing, or exaggerating their physical contributions to the task to make them visible and comprehensible (Clark & Krych, 2004).

In contrast, vertical transitions mark larger changes in the course of a joint activity and fall between its sub‐tasks. This implies that they prompt participants to shift the focus of their attention from the concurrent sub‐task and update the joint commitment between participants by agreeing to switch to the next sub‐task (Bangerter et al., 2022). It also implies that vertical transitions do not entail simultaneous physical contributions to the nonlinguistic task, potentially reducing the dual‐tasking load for participants. Hence, participants would have more resources to plan and produce (1) more lexicalized forms of coordination markers (e.g., *okay, yeah, all right, exactly, perfect, there you go, cool, well done*), (2) which are more likely to shift participants' attention to register the hierarchical changes in their activity. Unlike minimal markers of understanding, words like *okay* and *yeah* tend to be registered as markers introducing new contribution to the conversation and are more often followed by floor transfer than *uh‐huh* (Drummond & Hopper, 1993; Jefferson, 1984).

The evidence reviewed here raises the question of whether the periods of active engagement in a joint task (i.e., horizontal transitions) pose universal constraints on the form and lexicality of horizontal coordination markers, favoring the production of minimal tokens (*uh‐huh, mm*) and repetitions over more lexicalized words (*okay, all right*).

### 1.4 Current study

It has been previously established that specialized markers signaling attention and understanding (e.g., *uh‐huh*, *yeah*, *okay*) play an important role in helping participants ground each other's contributions and coordinate joint tasks. Previous studies have focused on markers produced only by active listeners (Dideriksen et al., 2023; Dingemanse et al., 2022; Fusaroli et al., 2017), analyzed the functions of a certain lexical form (Bangerter et al., 2024; Betz, Sorjonen, Mondada, & Deppermann, 2021), or documented the use of coordination markers in closely related languages (Bangerter & Clark, 2003). However, they lack the cross‐linguistic perspective needed to test how universal conversational infrastructure might shape the functional specialization of coordination markers for horizontal and vertical transitions. Robust tests of these claims would require a large sample of the world's languages, which, however, is incompatible with the depth of analysis required. An alternative is to investigate a limited sample of maximally diverse languages to simulate the grammatical diversity space of human languages (Stoll & Bickel, 2013), a strategy we adopt in the current study.

We investigated what words speakers of three maximally diverse languages (Swiss French, Vietnamese, and Shipibo‐Konibo) use to coordinate horizontal and vertical transitions in joint activities. As a joint activity task, we used a cooperative LEGO‐building paradigm (Clark & Krych, 2004) that had been previously analyzed for the production of coordination markers in English (Bangerter & Clark, 2003). Unlike spontaneous conversation, this task involved a clearly structured coordination routine, likely adopted by most participants, with a predictable hierarchy of horizontal and vertical transitions. Following the original paradigm, we tested participants in two conditions: visible (participants could see each other's actions through a transparent screen) and hidden (participants were completely separated by a barrier and had to rely just on verbal coordination). For each language, we manually annotated the conversations related to the task for both horizontal and vertical transitions. We tested whether (i) the production rates of coordination markers varied as a function of the participants' native language, sex, and familiarity (Section 3.1), (ii) if participants adjusted the production of coordination markers to their task roles and experimental conditions (Section 3.2). Lastly, we analyzed (iii) the distribution of coordination marker forms between horizontal and vertical transitions (Sections 3.3 and 3.4).

We predicted that (1) in each target language, participants use a set of specialized words as conventionalized coordination markers to navigate joint activities and that (2) these words are used as a system of transition‐specific markers to distinguish continuations of ongoing sub‐tasks (with markers specialized for horizontal transitions) from openings and closings of sub‐tasks (with markers specialized for vertical transitions). Further, we predicted that (3) repetitions and minimal markers (*mm, mm‐hm, uh‐huh*) might show stronger associations with horizontal transition contexts, while lexical markers (*all right, okay, perfect*) with vertical contexts, irrespective of language.

## 2 Methods

### 2.1 Target languages

Languages have evolved exhibiting a great diversity of phonological and morphosyntactic traits. However, certain aspects of this diversity might be constrained by universal functional pressures of interaction environments in the process of convergent evolution (Dingemanse et al., 2013). Therefore, studying how language use leads to convergent outcomes in different languages, it is important to compare languages that are most structurally different, that is, those with the greatest typological diversity, where similarities are least expected (Stoll & Bickel, 2013). The languages that we investigated here (Vietnamese, Swiss French, Shipibo‐Konibo) come from diverse genealogical taxonomies (Austroasiatic, Indo‐European, and Panoan families, respectively) and exhibit the maximum diversity in their typological traits (Stoll & Bickel, 2013).

In the domain of word formation, these languages use drastically different strategies of expressing morphosyntactic relations, which might potentially affect the kinds of linguistic markers (phonological, lexical, or grammatical) these languages employ to support communicative grounding in joint activity contexts. Vietnamese (Austroasiatic) is an analytic language with predominantly monomorphemic words and no inflectional morphology (Nguyễn, 1997). Grammatical and syntactic relations in Vietnamese are indicated through word order (Nguyễn, 1997) or separate “function words” (Thompson, 1965), and lexical meaning is also conveyed through six phonemic tones (Brunelle, 2015). Swiss French (Indo‐European) is a synthetic language that expresses morphosyntactic relations in a sentence through affixation, for example, verb conjugations and noun inflections (Judge & Healey, 1985). Morphemes in French are cumulative and may combine several grammatical categories (e.g., tense, person, and number) in a single morpheme (Judge & Healey, 1985), resulting in moderate morpheme‐per‐word ratios. In contrast, Shipibo‐Konibo (Panoan) is a synthetic language with complex polysynthetic tendencies of word formation. Compared to analytic Vietnamese and moderately synthetic French, words in Shipibo‐Konibo may contain multiple morphemes, expressing various aspects of grammatical, syntactic, and semantic meaning through affixation and phonetically bound enclitis (Valenzuela, 2003).

Given the large variation in how these languages express meaning, it remains unclear whether their speakers acknowledge transitions in a joint activity with lexical markers, for example, transition‐specific words such as *yeah, uh‐huh, okay* as in English, or if they mark acknowledgment grammatically via affixation (e.g., in Shipibo‐Konibo) or via distinct phonemic tones (e.g., in Vietnamese). Furthermore, these typological differences in morpheme‐per‐word ratios could affect the linguistic forms of lexical coordination markers.

### 2.2 Participants

We recorded a total of 182 sessions (*N* = 364 participants), 56 (*N* = 112) sessions in Vietnam, 43 sessions (*N* = 86) in Switzerland, and 83 sessions (*N* = 166) in Peru. Participants were tested in dyads of directors and builders in community spaces (university campus rooms in Vietnam and Switzerland and village community centers in Peru). Sixty‐six sessions were excluded from analyses due to equipment failure or camera view obstruction (*N* = 32), inconsistencies in the instruction procedure (*N* = 25), task rule violations (*N* = 6), extreme weather conditions (*N* = 2), and participation of a non‐native speaker (*N* = 1). The final sample included *N* = 232 participants (or 116 sessions), *N* = 80 in Vietnam, *N* = 78 in Switzerland, and *N* = 74 in Peru. All participants were native speakers of the target languages with no history of major speech‐ or hearing‐related medical conditions that could prevent them from participating in the study. Few participants in Switzerland (15.4%) and Peru (2.7%) reported minor difficulties with speaking (lisping or stuttering), reading (dyslexia), hearing, or the late onset of speaking in childhood.

In Vietnam, most participants were undergraduate students who were recruited and tested at a university in a large city in Northern Vietnam. Eighty participants (*N*_male_ = 40, *M_age_* = 19.5, *SD* = 1.36) were arranged into 10 male‐male, 10 female‐female, and 20 mixed‐sex dyads, with equal ratios of males and females in roles of director and builder. All participants were monolingual speakers of Vietnamese, 53.75% reported proficiency in at least one foreign language.

In Switzerland, most participants were undergraduate students who were recruited and tested at a university in a French‐speaking town in the west of Switzerland. Seventy‐eight participants (*N*_male_ = 40, *M_age_* = 22.24, *SD* = 2.65) were arranged into 10 male‐male, 9 female‐female, and 20 mixed‐sex dyads. Male and female participants were evenly distributed between the roles of directors (49% males vs. 51% females) and builders (54% males vs. 46% females). All participants were native speakers of French (73% monolingual), most reported proficiency in at least one foreign language (92.3%).

In Peru, participants were recruited and tested in three Shipibo‐Konibo villages (*Comunidades Nativas*) situated along the Ucayali river in the Peruvian Amazon. These three villages varied in their population sizes (from 150 to 1000 residents) and their proximity to urban capital Pucallpa. Seventy‐four participants (*N*_male_ = 37, *M_age_* = 25, *SD* = 5.59) were arranged into 9 male‐male, 9 female‐female, and 19 mixed‐sex dyads. Distribution of males and females between participant roles was comparable in the roles of director (51% males vs. 49% females) and builder (49% males vs. 51% females). All participants were native speakers of Shipibo‐Konibo and bilingual in Spanish.

The study was approved by the Ethics Committee of the Faculty of Arts and Social Sciences at the University of Zurich (Agreement Nr. 21.9.13) and by the Research Ethics Commission of the University of Neuchâtel (Agreement Nr. 87‐2021). All participants provided written informed consent and were compensated monetarily.

### 2.3 Data collection

Data collection lasted from December 2021 until January 2023. In the beginning of data collection, we implemented COVID‐19 safety measures (see Supporting Information S1, Section 3) to protect our study participants in Vietnam (*N* = 18 dyads or 45%). One of these measures required participants to wear medical masks provided by the research team. As masks partially impaired the use of nonverbal communicative signals, we checked whether participants might have overcompensated this loss with increased production of verbal coordination markers. We compared whether the average rates of coordination marker production in Vietnamese participants changed if we excluded the masked participants. There were no differences in coordination marker production rates between the masked and unmasked samples.

#### 2.3.1 Experimental setup

**Fig. 1:**
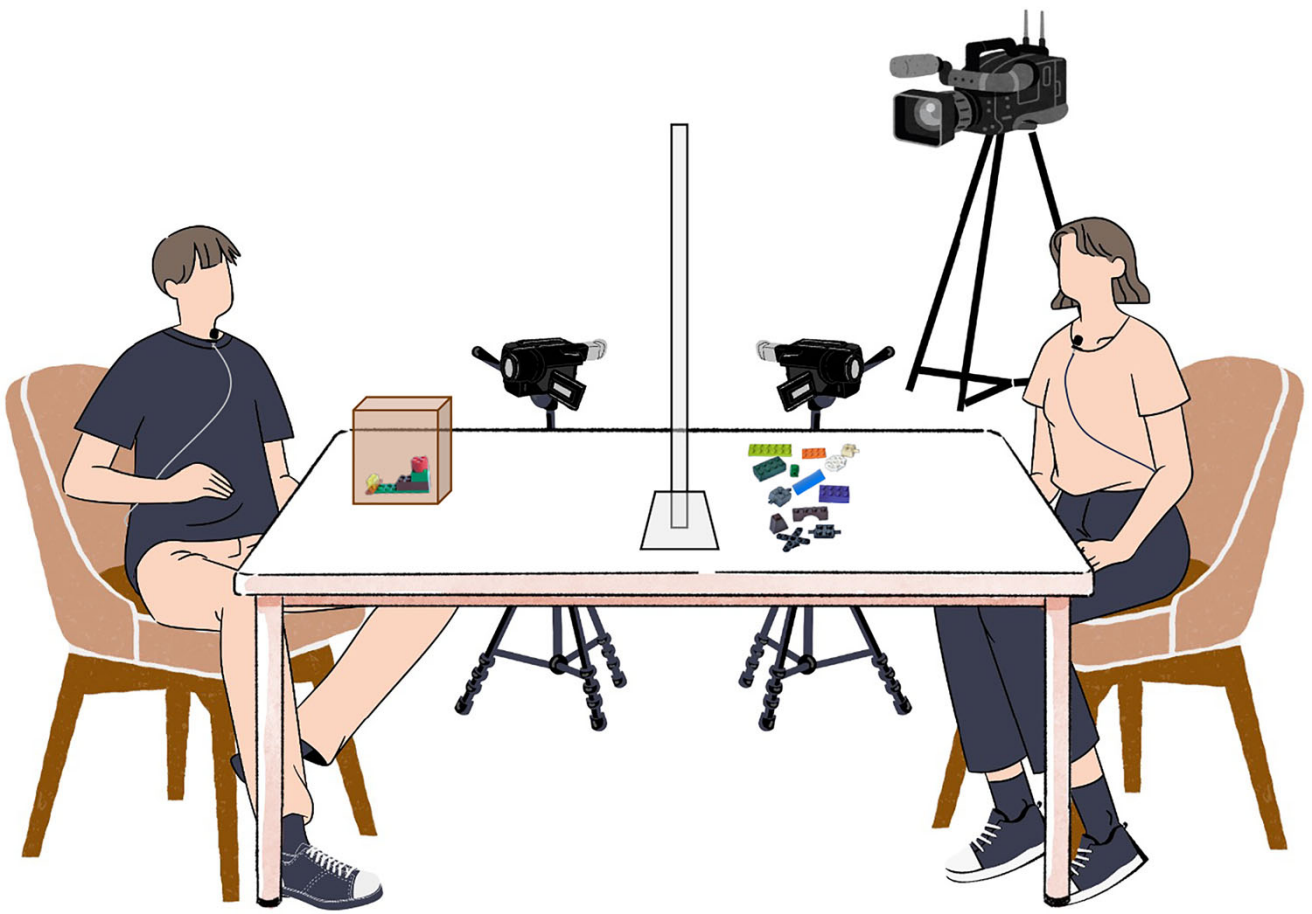
Illustration of the working spaces of director (on the left) and builder. Participants are recorded with two lavalier microphones and three video cameras.

We used the LEGO‐building paradigm (Clark & Krych, 2004; see Figure 1) as a joint activity task, during which dyads of directors and builders cooperatively constructed 10 LEGO models in visible (no barrier) and hidden (fully separated by a barrier) conditions. Participants sat on opposite sides of a table, facing each other. A transparent COVID‐safety screen was placed between participants, separating the table into individual working spaces. In the directors' working space, there was an empty opaque box for concealing prototype LEGO models. In the builders' working space, 79 unique LEGO Classic bricks were arranged by color (to minimize potential noise of moving blocks around). Fifteen out of 79 blocks were distracting pieces; the rest were to be used to complete the models. Each prototype model consisted of six blocks assembled into abstract arrangements of four blocks high (stimuli pictures are provided in Supporting Information S1, Section 2).

Participants were recorded with three video cameras. Two wide‐angle Zoom Q8 cameras were installed in participant's working spaces, capturing a clear view of their facial expressions and model construction process. They were equipped with native external microphones or with SGH‐6 shotgun microphones. The third camera, SONY PXW‐Z90, was set approximately 1.5 meters away from the table and captured both participants and their working spaces in the same frame. Lavalier Sennheiser AVX‐MKE2‐3 microphones were attached to each participant and connected to the SONY camera via transmitters.

#### 2.3.2 Procedure and instructions

Trained native speakers conducted the experiments, explaining the study to participants, administering consent forms and self‐report questionnaires, supervising experimental sessions, and debriefing participants. Each session began with an instruction phase, where the experimenter explained the task rules in participants' native language. Directors were given unique prototype models, concealed in a box placed in the director's working space. Their task was to instruct builders how to assemble a model identical to the prototype, without revealing the prototype. Builders then constructed models using the blocks placed in their working space. There was no limit on construction time, and participants were allowed to converse as much as they needed. For all dyads, trials were held in visible and hidden conditions. In the visible condition, participants could see each other fully through the transparent screen. They were allowed to point and gesture, although we did not explicitly instruct them to do so. In the hidden condition, the screen between participants was covered with an opaque cloth, and participants were asked to remain in their working space. They were not allowed to point, gesture, or show model blocks over the screen.

Next, participants practiced to build one training model. Once the experimenter made sure the instructions were clear and the participants had no further questions, the experiment began. The experimenter stayed in the testing room to supervise the change of trials and observe the participants for rule violations. After each trial, the experimenter compared the models and explained any inconsistencies with respect to the color, shape, or position of the blocks. Dyads built five consecutive models in each condition (one model per trial), and then switched conditions (the order of conditions was counterbalanced). The model order remained unchanged regardless of condition order.

### 2.4 Data annotation

We used ELAN v.6.3 (Wittenburg, Brugman, Russel, Klassmann, & Sloetjes, 2006) to manually segment, transcribe, translate, and annotate experimental sessions. The participants' speech was segmented into utterances following intonation boundaries (*N*_total_ = 110,614) and included only task‐related utterances exchanged between participants (*N*_fr_ = 28,223, *N*_vi_ = 35,077, *N*_shp_ = 47,314). Each speech segment was annotated for experimental setting (condition, model number), corresponding sub‐task (phase, sub‐phase, block), question‐answer sequences, and transition type (horizontal or vertical). We manually extracted coordination markers and their respective transition labels from the annotated speech segments, for example, *okay*_vertical_ or *okay*_horizontal_.

#### 2.4.1 Sub‐components of the joint task

**Fig. 2:**
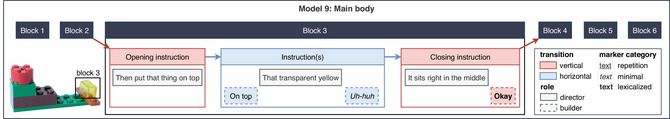
Hierarchical representation of a joint task (model 9), its sub‐tasks (blocks), and sub‐subtasks (instructions for block 3) in the LEGO‐building activity. Each speech segment produced by directors and builders was annotated for vertical (red) or horizontal (blue) transition contexts. Coordination markers received transition labels corresponding to their respective speech segments (e.g., repetition_horizontal_, *uh‐huh*_horizontal_, *okay*_vertical_) and were annotated for marker category (repetition, minimal, or lexicalized).

Each six‐block LEGO model was treated as a separate joint task with corresponding block sub‐tasks (*block 1, block 2*, etc.) and sub‐subtasks of individual block instructions (e.g., *opening instruction, closing instruction*, see Figure 2). We assigned unique labels to each block in order of their appearance, annotating which block participants placed in the given moment (e.g., *block 3*). We coded the change of blocks once participants agreed to move to the next block (e.g., *block 4*) or decided to return to one of the previous blocks (e.g., *block 2*). If participants worked on several blocks simultaneously, such speech segments were annotated for all respective blocks (e.g., *block 2, block 3*).

Some dyads went through additional sub‐components of their joint activity, that is, openings (*Entry*) and closings (*Exit*) of trials, during which participants established and dissolved the commitment to engage in the task. Entries and Exits were treated as higher‐order sub‐components of the task with vertical transitions into and out of the *Main body* of the task, that is, construction‐oriented dialogue. In addition, dyads occasionally engaged in extra pre‐construction (*Block identification*) and post‐construction (*Block check*) coordination routines. If present, these additional routines were excluded from further analyses; we provide their detailed descriptions and coding conventions in Supporting Information (S1, Section 1).

#### 2.4.2 Transitions

We coded *N* = 91,274 transitions based on whether participants were elaborating on a specific sub‐task of their activity (*horizontal*) or moving to the next sub‐task (*vertical*). In the hidden condition, directors and builders had to engage in extended negotiations of closing one block and moving to the next. Hence, often attempts to make a vertical transition into the next block were ignored and followed by more elaborate instructions for the current block. We thus determined that true vertical transitions occurred with the physical change of blocks, that is, vertical transition sequences started with the very last informative instruction for the current block (e.g., *block 4*) and ended with the first informative instruction for the next block (*block 5*). Transition sequences indicating openings and closings of trials (*Entry* — *Exit*), changes in coordination routines (*Block Identification* — *Block Placement* — *Block Check*), and changes of block sub‐tasks within the main coordination routine (*block 1* — *block 2* — etc.) were coded as vertical (*N* = 33,468). Transition sequences within block sub‐tasks were coded as horizontal (*N* = 57,806).

#### 2.4.3 Extraction of coordination markers

Coordination markers were extracted from Entries, Exits, and the main task coordination routine. In this study, we focused on generic lexical markers (words like *yes*, *yeah*, *uh‐huh*, *okay*, *exactly*, or repetitions) that ratified the contributions of participants to the joint task and acknowledged the progress toward the joint goal. Some of these words, however, could be used to express agreement in affirmative responses to questions, for example, D: *Does this block fit in the middle?* B: ***Yeah***. To separate coordination markers from agreement markers, we coded speech segments for *questions* and *answers*. In few instances, when participants used coordination markers like *okay?*, *all right?*, *yeah?* as interrogative words to elicit acknowledgment from the other participant, we extracted them as coordination markers, for example, D: *And then you place this block in the middle*, ***okay*** *?* If such utterances successfully elicited an identical marker, for example, B: ***Okay***, *done*, we also extracted such *okay* as a coordination marker. Other types of markers that appeared in **answer**‐tagged segments were considered agreement markers and thus excluded from further analyses.

Repetitions have also been found to indicate the need for communicative repair, via so‐called restricted offers (Dingemanse et al., 2015; Fusaroli et al., 2017) or trouble‐presenting repeats (Dingemanse, Blythe, & Dirksmeyer, 2014). To avoid mixing repetitions used as coordination markers with repair markers, we did not extract repetitions from **question**‐tagged speech segments. Since such repair requests might not contain explicit question markers, we relied on the intuition of our transcribers to indicate if an utterance was marked as a question by means of prosody.

Coordination markers are not the only linguistic conventions that serve to maintain the common ground between participants and track their progress through a joint task. For example, participants might use explicit, agreement‐eliciting interrogative words such as *correct?* or *c'est tout bon?* in Swiss French, *đúng không?* in Vietnamese, and *ikon?* in Shipibo‐Konibo. Grounding might also be achieved via grammatical markers of affirmation, for example, verb suffixes *‐í* and *‐kin* in Shipibo‐Konibo (Valenzuela, 2003). Lastly, joint action coordination is also supported by nonverbal behavioral cues, for example, body proximity and orientation, gaze, laughter (Heesen, Genty, Rossano, Zuberbühler, & Bangerter, 2017), head nods, eye blinks (Hömke, Holler, & Levinson, 2018), and sensorimotor communication (Vesper et al., 2017). Here, we focused only on lexical coordination markers, thus excluding all these other coordinating conventions.

#### 2.4.4 Classifying markers as repetitions, minimal, and lexicalized markers

Lastly, we classified extracted coordination markers into the categories of minimal forms, repetitions, and lexicalized forms. Building on the existing classifications of response markers (Iwasaki, 1997; Ward, 2006), we defined minimal markers as intentionally produced vocalizations that are directed at other interlocutors, have no referential meaning, and show no syntactic correspondences, for example, *mm*, *mm‐hm*, and *uh‐huh*. Repetitions, also known as substantive backchannels (Iwasaki, 1997), included smaller installments (partial repetitions), verbatim displays (full repetitions; Clark & Schaefer, 1989), restatements, and summary statements produced to ground the previous speaker's contributions. Other forms of coordination markers were considered lexicalized markers, for example, generic words like *yeah*, *yes*, *right*, *okay* that additionally express acceptance and agreement and markers like *perfect*, *exactly*, *that's right*, *well done* that express assessment.

#### 2.4.5 Coding reliability

Inter‐rater reliabilities were calculated between the first author and three trained second coders (one per target language) that were naive to the research questions of our study. For Vietnamese and Swiss French sessions, second coders were native speakers who worked directly with speech transcripts. For Shipibo‐Konibo sessions, the second coder was a native speaker of Peruvian Spanish who worked with translated speech segments. We double‐coded around 10% (*N* = 11,615) of utterances, or 13 sessions, from all experimental recordings (*N*_total_ = 119,434). Per language, we double‐coded four Vietnamese sessions (or 9.2%, *N* = 3212 utterances), four Swiss French sessions (or 9.8%, *N* = 2965), and five Shipibo‐Konibo sessions (or 10.1%, *N* = 5438). Sessions contained pre‐segmented participant speech. Each pre‐segmented utterance was assessed as whether it occurred outside of experimental trials (no annotation), was addressed to research stuff (no annotation), or was produced during a trial and addressed to the other participant (annotated accordingly). Inter‐rater reliability check returned good global agreement estimates for each language (Cohen's $\kappa_{vi}=0.95$, $\kappa_{fr}=0.95$, $\kappa_{shp}=0.95$). Annotation categories and their inter‐rater agreement estimates are provided in Table 1.

**Table 1:** Annotation scheme and inter‐rater reliability scores for Vietnamese (κ_vi_), Swiss French (κ_fr_), and Shipibo‐Konibo (κ_shp_) transcripts

IRR scores (Cohen's κ)			ELAN annotations		
κ_vi_	κ_fr_	κ_shp_	Category	Labels	Description
0.99	1.00	0.99	Model	mod1, …, mod10	Model number
0.98	0.99	0.98	Condition	visible, hidden	Experimental condition
0.90	0.84	0.91	Phase	Entry, Main body, Exit	Trial opening, model building, and trial closing
0.93	0.90	0.90	Sub‐phase	Block ID 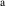 , Block PL, Block CH 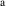	Task coordination routines
0.90	0.96	0.90	Block	b1, …, b6	Block labels in order of appearance
0.82	0.83	0.83	Transition	horizontal, vertical	Transitions within or between task sub‐components
0.88	0.82	0.83	Q&A	q, a, none	Questions (q), answers (a)

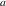
 Block ID (pre‐construction selection of all model blocks) and Block CH (post‐construction check of model configuration) sub‐phases were distinguished from Block PL (consecutive identification and placement of blocks) and excluded from further analyses due to uncertainties in their transition hierarchy.

### 2.5 Bayesian analyses

To test our research questions, we fitted mixed‐effects Bayesian regression models^1^ using brms v.2 20.4 (Bürkner, 2017) package in R 4.3.1 (R Core Team, 2023). For each model, we specified weakly informed priors. All models were run using four Markov Chain Monte Carlo chains (MCMC), with a total of 10,000 iterations per chain and first 2000 iterations as warm‐up. Each model returned 32,000 posterior samples. Model convergence was assessed using convergence statistics ($\hat{R}\leq 1.01$), absence of divergent transitions, and visual inspection of trace plots. Model selection, if necessary, was carried out based on Pareto‐smoothed importance sampling or PSIS LOO (Vehtari, Gelman, & Gabry, 2017) and expected log‐predictive density (ELPD). To test our research questions, we computed posterior predicted contrasts between the group means (Δ) and interpreted the results based on 95% credible intervals (CI) from predictions of the posterior and the probability of direction index (*pd*). *Pd* was provided when credible intervals intersected with 0; we interpreted *pd* coefficients as the degree of certainty that a specific parameter had a positive or negative effect on the response variable.

In what follows, we first test whether the rates of coordination marker production were affected by participants' language, sex, and their familiarity with each other (Section 3.1) and how participants adapted the use of coordination markers to their designated task roles (director vs. builder) and the changes in the interaction environment (visible vs. hidden condition) in Section 3.2. In Section 3.3, we address our predictions regarding whether participants use distinct linguistic forms of coordination markers to distinguish horizontal transitions from vertical. Lastly, we explore how repetitions, minimal, and lexicalized categories of coordination markers were overall distributed between horizontal and vertical transition contexts and whether these distribution patterns were observed in each target language (Section 3.4).

## 3 Results

We analyzed the total *N* = 91,274 utterances (or 2522.11 min of interaction time) coded for transitions (*N*_vi_ = 27,393 utterances or 796.90 min, *N*_fr_ = 24,151 utterances or 767.64 min, *N*_shp_ = 39,730 or 957.57 min). Overall, 30% of the utterances (*N* = 27,801) contained coordination markers, 38.5% (*N* = 9302) in Swiss French data subset, 29% (*N* = 8050) in Vietnamese, and 26% (*N* = 10,449) in Shipibo‐Konibo. We extracted *N* = 32,523 coordination markers (*N*_vi_ = 9602, *N*_fr_ = 11,193, *N*_shp_ = 11,728).

On average, participants produced 12.2 (*SD* = 2.94) coordination markers per minute of interaction time in Vietnamese, 14.94 markers (*SD* = 3.63) per minute in Swiss French, and 12.64 markers (*SD* = 4.0) per minute in Shipibo‐Konibo. 69.9% (*N* = 22,744) of coordination markers were produced in the hidden condition versus 30.1% (*N* = 9779) in the visible condition trials. Similar distributions between hidden versus visible conditions were observed within each language subset, with 71% (*N* = 6822) versus 29% (*N* = 2780) in Vietnamese, 68% (*N* = 7656) versus 32% (*N* = 3537) in Swiss French, and 70.5% (*N* = 8266) versus 29.5% (*N* = 3462) in Shipibo‐Konibo.

### 3.1 Variation in coordination marker production rates by language, familiarity, and sex

Previous research shows that the amount of verbal grounding feedback differs across cultures and languages (Clancy, Thompson, Suzuki, & Tao, 1996; Li, 2006; Maynard, 1986; Stubbe, 1998; Tao & Thompson, 1991; White, 1989), between strangers and friends (Jucker & Smith, 1998), and is influenced by the sex of the interlocutors (Bilous & Krauss, 1988; Hirschman, 1994; Jenkins & Cheshire, 1990; Mulac, Wiemann, Widenmann, & Gibson, 1988; Mulac & Bradac, 2012; Roger & Schumacher, 1983; Roger & Nesshoever, 1987; Zimerman & West, 1975). However, some studies did not find evidence supporting cross‐linguistic differences in grounding feedback (Dideriksen et al., 2023) and sex groups (Dixon & Foster, 1998; Stubbe, 2013). Our study thus re‐examines the effects of language, sex, and familiarity on the production of words such as *yeah, uh‐huh, okay* as joint action coordination markers.

#### 3.1.1 Model specifications

We aggregated a subset of data containing coordination marker counts produced in each experimental trial per dyad (i.e., 10 data entries per dyad), speech utterance counts per trial, dyad ID, language (Swiss French, Vietnamese, or Shipibo‐Konibo), dyadic sex group (male‐male, female‐female, or mixed), and familiarity (less familiar vs. more familiar). Familiarity scores were recoded as a dichotomous factor based on the mean Likert scores per dyad. Scores ranging from 0 to 3 (equivalent to *not familiar, slightly familiar, somewhat familiar*) were coded as 0 or *less familiar* dyads; scores from 3.5 to 5 (equivalent to *moderately familiar* and *extremely familiar*) were coded as 1 or *more familiar* dyads. After recoding, the distribution of less familiar to more familiar dyads in each language sample was 55% versus 45% in Vietnamese, 69% versus 31% in Swiss French, and 30% versus 70% in Shipibo‐Konibo.

We fitted model 1.1 as a mixed‐effects negative binomial regression. As response variable, we set coordination marker counts, normalized by utterance counts through rate argument. As fixed‐effect predictors, we set language, dyadic sex group, and familiarity. As random intercept, we set dyad ID. We analyzed the total of *N* = 1159 data points (one trial from the Vietnamese data sample was excluded for the exposure of the model prototype).

(1.1) $$\begin{array}{cc} \mathrm{marker}\;{\mathrm{count}}_{i} & ∼\mathrm{Negative}\;\mathrm{Binomial}(\mu_{i},\alpha )\\ \log\left(\mu_{i}\right)\;|\;\mathrm{rate}\;\left(n_{\mathrm{utterance}}\right) & =\beta_{0}+\beta_{1}{\mathrm{language}}_{i}+\beta_{2}{\mathrm{sex}}_{i}+\\ & \;+\beta_{3}{\mathrm{familiarity}}_{i}+\left(1\;|\;\mathrm{dyad}\;\mathrm{ID}\right) \end{array}$$

As model 1.1 estimated general effects of sex and familiarity on coordination marker production (irrespective of language), we fitted model 1.2 with an interaction between each predictor and participants' language. Model 1.2 predicted the changes in coordination marker production as a function of participant sex and familiarity, for each language individually:

(1.2) $$\begin{array}{cc} \log\left(\mu_{i}\right)\;|\;\mathrm{rate}\;\left(n_{\mathrm{utterance}}\right) & =\beta_{0}+\beta_{1}{\mathrm{language}}_{i}+\beta_{2}{\mathrm{sex}}_{i}+\beta_{3}{\mathrm{familiarity}}_{i}+\\ & +\beta_{4}\left({\mathrm{language}}_{i}\times {\mathrm{sex}}_{i}\right)+\beta_{5}({\mathrm{language}}_{i}\times \\ & \times {\mathrm{familiarity}}_{i})+\left(1\;|\;\mathrm{dyad}\;\mathrm{ID}\right) \end{array}$$

For both models, we specified weakly informed priors. The mean value and standard deviation of the intercept were set to $\beta_{0}∼$ Normal (μ=0,σ=3.7) markers per trial. The prior distribution reflected the average number of markers produced by a dyad (*N* = 40) given the average number of transitions per single block model (*N* = 3), that is, approximately *N* = 18 transitions plus opening and closing transitions (*N* = 2) per participant and per model. We set priors that were symmetrical and centered on zero, thus uninformative with respect to the direction of the effect for language, participant sex, and familiarity $\beta_{1},\beta_{2},\beta_{3}∼$ Student_t_ (ν=3,μ=0,σ=1.5). Shape parameter was set to α∼ Gamma(0.01,0.01).

#### 3.1.2 Effects of language

Our findings provide strong evidence that the average production rates of coordination markers vary across languages (Figure 3.1). The highest production rate per trial was estimated for Swiss participants (*M*_rate_ = 35.70), and it substantially exceeded the average production rates in Vietnamese (Δ= 8.72, 95% CI [5.29, 12.18], *M*_rate_ = 26.98) and Shipibo‐Konibo participants (Δ= 12.73, 95% CI [9.36, 16.16], *M*_rate_ = 22.97). The average production rate of Vietnamese participants, in turn, exceeded that of Shipibo‐Konibo participants (95% CI [1.29, 6.76]). Overall, Swiss participants used the highest number of lexical coordination markers per trial, followed by Vietnamese participants, with Shipibo‐Konibo participants using the fewest.

**Fig. 3:**
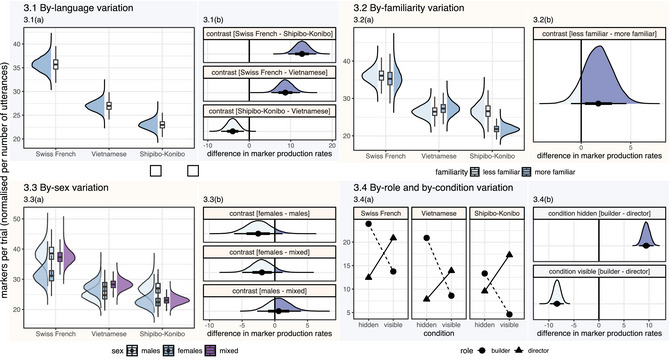
Variation in the production rates of coordination markers by participant language (3.1), familiarity (3.2), and sex (3.3), participant role and experimental condition (3.4). Posterior predicted variation in coordination marker production within each target language (a) and language‐independent contrasts between their group means (b).

#### 3.1.3 Effects of dyad familiarity

First, we tested the general effects of participant familiarity on the production of coordination markers, irrespective of language (Figure 3.2). Model 1.1 estimated that less familiar dyads (*M*_rate_ = 29.42) produced slightly more markers per trial than more familiar dyads (95% CI [–1.01, 4.58], *M*_rate_ = 27.68). However, this effect was weak as the increase in production rates was estimated with 89.01% probability (*pd*). Model 1.2 revealed that strong effects of familiarity were observed only in Shipibo‐Konibo participants (Δ = 4.83, 95% CI [0.25, 9.24], *M*_rate_ = 26.65).

#### 3.1.4 Effects of participant sex

We found only weak effects of participant sex on coordination marker production (Figure 3.3). Overall, female dyads produced on average 27.05 markers per trial, which was lower than the production rates in male (Δ =−2.55, 95% CI [−6.20, 1.21], *pd* = 91.19%, *M*_rate_ = 29.60) and mixed‐sex dyads (Δ =−1.95, 95% CI [−5.01, 1.17], *pd* = 89.45%, *M*_rate_ = 29.00). Model 1.2 estimated similar trends in Swiss participants, where female dyads (*M*_rate_ = 31.22) produced marginally less markers per trial than male dyads (Δ = −7.42, 95% CI [−15.18, 0.09], *pd* = 97.37%, *M*_rate_ = 38.65) and mixed dyads (Δ = −6.17, 95% CI [−12.36, 0.11, *pd* = 97.32%, *M*_rate_ = 37.39). In Shipibo‐Konibo female dyads, the average number of produced coordination markers (*M*_rate_ = 22.50) was lower than in male dyads (Δ =−4.61, 95% CI [−10.25, 0.97], *pd* = 94.98%, *M*_rate_ = 27.11) and did not differ from mixed‐sex dyads. In Vietnamese participants, however, female dyads (*M*_rate_ = 27.61) produced slightly more markers than male dyads (Δ = 2.81, 95% CI [−2.91, 8.43], *pd* = 84.03%, *M*_rate_ = 24.80) and about the same amount as mixed dyads.

**Fig. 4:**
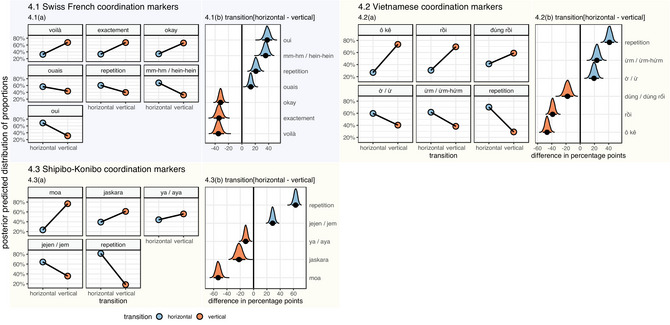
By‐transition distribution of coordination markers in Swiss French (4.1), Vietnamese (4.2), and Shipibo‐Konibo (4.3). Posterior predicted proportions of coordination markers between horizontal and vertical transition contexts (a) and contrasts between their predicted distributions (b).

**Fig. 5:**
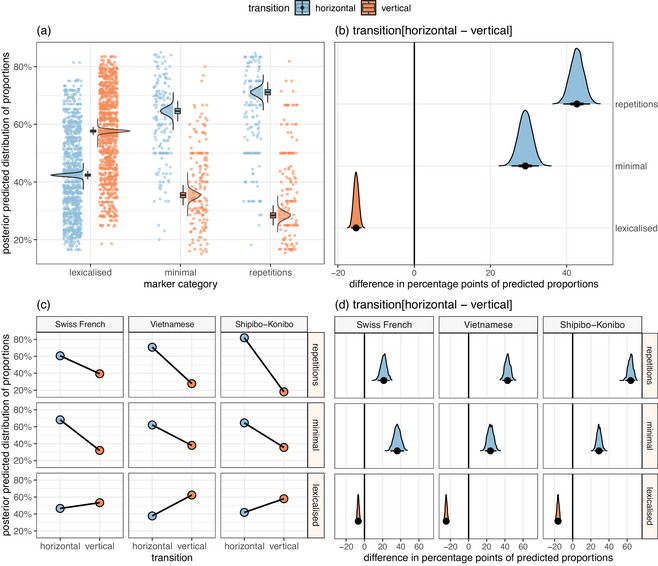
**Model 6.1** (a, b): Posterior predicted proportions of marker categories between horizontal and vertical transition contexts (plotted against the observed values) (a) and contrasts between their predicted distributions (b). **Model 6.2** (c, d): By‐language proportional distributions of marker categories (c) and by‐language contrasts between their predicted distributions (d).

Our models did not predict strong effects of participant sex on the production rates of coordination markers. However, male and mixed dyads tended to use marginally more coordination markers than female dyads. This tendency was observed in Swiss and (partially) in Shipibo‐Konibo participants, but not in Vietnamese participants.

### 3.2 Variation in coordination marker production rates by participant role, experimental condition, and language

Next, we tested whether speakers of each target language attuned their use of coordination markers to their respective roles and experimental conditions. In the visible condition, grounding between participants should be achieved at lower communicative costs. Directors could monitor the construction process and directly ratify or correct builders' contributions to the model construction. Builders, in turn, could ground directors' instructions nonverbally, that is, with pointing, nodding, display gestures, and mutual gaze (Clark & Schaefer, 1987). However, the loss of copresence and visibility in the hidden condition would be associated with higher grounding costs for builders (Clark & Brennan, 1991), which would lead to an increase in the production of verbal coordination markers to acknowledge directors' instructions (Bangerter & Clark, 2003). We, therefore, expect that the change of conditions from visible to hidden condition would be associated with a general increase in coordination marker production, specifically in builders.

#### 3.2.1 Model specifications

We compiled a new dataset containing coordination marker counts per participant per experimental trial (i.e., 10 data entries per participant, 20 entries per dyad), cumulative speech utterance counts per dyad per trial, participant ID, dyad ID, language (Swiss, Vietnamese, or Shipibo‐Konibo), participant role (director or builder), and experimental condition (visible or hidden). We fitted model 2 as a mixed‐effects negative binomial regression. As response variable, we set marker counts, normalized per utterance counts. As fixed‐effect predictors, we specified a three‐way interaction among language, participant role, and condition. As random effects, we nested participant ID within dyad ID. In total, we analyzed *N* = 2318 data points.

(2) $$\begin{array}{cc} \mathrm{marker}\;{\mathrm{count}}_{i} & ∼\mathrm{Negative}\;\mathrm{Binomial}(\mu_{i},\alpha )\\ \log\left(\mu_{i}\right)\;|\;\mathrm{rate}\;\left(n_{\mathrm{utterance}}\right) & =\beta_{0}+\beta_{1}{\mathrm{language}}_{i}+\beta_{2}{\mathrm{role}}_{i}+\beta_{3}{\mathrm{condition}}_{i}+\\ & +\beta_{4}\left({\mathrm{language}}_{i}\times {\mathrm{role}}_{i}\right)+\beta_{5}({\mathrm{language}}_{i}\times \\ & \times {\mathrm{condition}}_{i})+\beta_{6}\left({\mathrm{role}}_{i}\times {\mathrm{condition}}_{i}\right)+\\ & +(1\;|\;\mathrm{dyad}\;\mathrm{ID}/\mathrm{participant}\;\mathrm{ID}) \end{array}$$

As weakly informed priors, we lowered the intercept deviation to $\beta_{0}∼$ Normal (μ=0,σ=3) as we expected that participants might use less coordination markers in visible condition. We kept zero‐centered, symmetrical predictions for language, participant role, and condition $\beta_{1},\beta_{2},\beta_{3}∼$ Student_t_ (ν=2.5,μ=0,σ=2.5).

#### 3.2.2 Effects of participant role and experimental condition

In all language subsets, participants adjusted their coordination strategies between the visible and the hidden conditions (Figure 3.4). Model 2 revealed that builders (*M*_rate_ = 19.37) produced more coordination markers in the hidden condition than directors (Δ = 9.43, 95% CI [8.01, 10.88], *M*_rate_ = 9.94). We observed this trend in Swiss (Δ = 11.46, 95% CI [8.54, 14.54]), Vietnamese (Δ = 13.08, 95% CI [10.61, 13.08]), and Shipibo‐Konibo participants (Δ = 3.77, 95% CI [1.95, 5.62]). With the change of condition from hidden to visible, the production rate of builders (*M*_rate_ = 8.97) substantially decreased compared to those of directors (Δ = −8.38, 95% CI [−9.78, −7.00], *M*_rate_ = 17.34). We found strong evidence supporting this difference in Swiss (Δ = −7.13, 95% CI [−10.20, −4.15]), Vietnamese (Δ = −5.33, 95% CI [−7.37, −3.37]), and Shipibo‐Konibo participants (Δ = −12.67, 95% CI [−14.81, −10.64]).

Irrespective of the experimental condition, the role of the builder predicted higher rates of marker production in Swiss (Δ = 2.17, 95% CI [−0.61, 4.98], *pd* = 92.15%) and Vietnamese participants (Δ = 3.87, 95% CI [1.91, 5.98]). However, in Shipibo‐Konibo participants, we observed the opposite trend, where the role of the builder predicted a decrease in marker production rates compared to directors (Δ =−4.45, 95% CI [−6.23, −2.65]). Overall, builders produced more coordination markers than directors in the hidden condition, while directors used more markers than builders in the visible condition. We found strong evidence suggesting that such a division of grounding contributions occurred in all target languages.

### 3.3 Variation in proportional distributions of coordination markers between horizontal and vertical transitions in each target language

In addition, we tested how the transition context influences the choice of linguistic forms of coordination markers. Previous findings suggest that the words *yeah, uh‐huh, okay* are used as specialized markers for distinct transition contexts (Bangerter & Clark, 2003), for example, horizontal *yeah* versus vertical *okay*. We thus hypothesized that coordination markers in other languages are also used contrastively, that is, to distinguish between horizontal and vertical transitions in joint activities. Specifically, we inferred whether the proportional distributions of various coordination markers substantially differed between horizontal and vertical transition contexts.

#### 3.3.1 Model specifications

For each language, we fitted separate models 3, 4, 5 with a beta‐binomial distribution. We aggregated new datasets containing distinct linguistic forms of coordination markers (e.g., *okay*, *uh‐huh*) and their count distributions in each transition contexts (e.g., *okay_horizontal_* vs. *okay_vertical_*) per participant. Each data point contained marker ID (two entries per marker per participant), its counts π in a specific transition context (e.g., *okay_horizontal_*), total counts of the marker form in both transition contexts $n_{markers\;total}$ (i.e., aggregated counts from *okay_horizontal_* and *okay_vertical_*), and participant ID.

(3, 4, 5) $$\begin{array}{cc} \mathrm{markers}\;\mathrm{per}\;{\mathrm{transition}}_{i} & ∼\mathrm{Beta}\;\mathrm{Binomial}(n_{markers\;total},\pi_{i},\phi )\\ \mathrm{logit}\left(\pi_{i}\right)\;|\;\mathrm{trials}\;\left(n_{\mathrm{markers}\;\mathrm{total}}\right) & =\beta_{1}\mathrm{marker}\;{\mathrm{form}}_{i}+\beta_{2}{\mathrm{transition}}_{i}+\\ & +\beta_{3}\left(\mathrm{marker}\;{\mathrm{form}}_{i}\times {\mathrm{transition}}_{i}\right)+\\ & +(1\;|\;\mathrm{participant}\;\mathrm{ID}) \end{array}$$

We set weakly informed priors for $\beta_{1}$, $\beta_{2}$ ∼ Normal (μ=0,σ=2) and shape parameter ϕ∼ Gamma (0.01,0.01). Model 3 was fitted for Swiss French (*N* = 910), model 4 for Vietnamese (*N* = 878), and model 5 for Shipibo‐Konibo (*N* = 690). The contrasts between distributions of individual marker forms in horizontal versus vertical transitions were calculated by subtracting 12,000 posterior predicted draws per condition. Contrasts were visualized and color‐coded to indicate the direction of the predicted effect; negative effects (red) represented a stronger association with vertical transitions, while positive effects (blue) indicated a stronger association with horizontal transitions.

#### 3.3.2 Vertical and horizontal markers in Swiss French

Forms of coordination markers and their phonetic transcriptions are provided in Table 2. In Swiss French, we distinguished 10 linguistic forms of coordination markers (*N* = 11,193): *ouais* (“yeah,” 36.5%), *OK* (“okay,” 26.3%), *mm‐hm / mm / hein‐hein* (“mm, uh‐huh,” 8.1%), repetitions or restatements (8%), *oui* (“yes,” 6.3%), *voilà* (“there you go,” 5.9%), *exactement / exact* (“exactly,” 4.8%), *parfait* (“perfect,” 1.6%), *d'accord* (“okay,” 1.3%), and *other* (1.1%). Category *other* (*N* = 123) encompassed low‐frequency words, predominantly assessment markers such as *top / tip top* (*N* = 38), *magnifique* (“magnificent, wonderful,” *N* = 23), *super* (*N* = 26), *effectivement* (“indeed,” *N* = 3), *tac* (“check,” *N* = 2), *correct* (*N* = 1). For further analysis, we selected a subset of most frequent linguistic forms *ouais*, *OK*, *mm‐hm / mm / hein‐hein*, repetitions, *oui*, *voilà*, and *exactement*. They constituted 95.9% (*N* = 10,740) of all extracted markers.

**Table 2:** Coordination marker forms (arranged by frequency) and their phonetic transcriptions provided in IPA

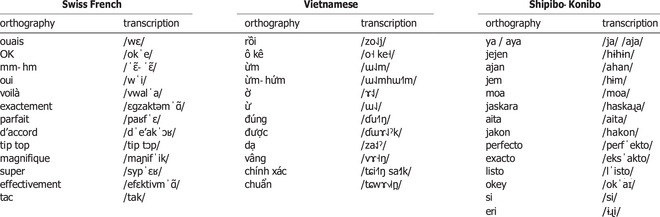

Some forms of coordination markers were produced predominately as stand‐alone markers, for example, acknowledgment tokens *mm‐hm / mm / hein‐hein* (96.8%), *oui* (64.3%), and *d'accord* (62.2%), and assessment tokens *top / tip top* (89.3%), *super* (71.4%), *magnifique* (66.7%), *parfait* (63.4%). Marker *voilà*, in contrast, was deployed mainly within turns (62.5%), that is, either as turn‐prefacing or turn‐final marker. When participants used a combination of several marker forms (e.g., *ouais ouais exactement OK*), they tended to appear within a speaking turn (64.6%). Other marker forms were distributed somewhat equally between stand‐alone and within‐turn contexts; detailed distributions of each marker form between stand‐alone and within‐turn contexts are provided in Supporting Information (S1, Section 4.1). The distribution of each marker form between the roles of builder and director are provided in Section 5.1 of S1.

Model 3 revealed that all linguistic forms were specialized either for horizontal or vertical transitions (Figure 4.1). Markers *voilà*, *exactement / exact*, and *OK* were consistently used to mark vertical changes. Hence, horizontal contexts predicted a decrease in the proportional distributions of *voilà* (Δ = −35.05, 95% CI [−43.00, −26.63]), *exactement* (Δ = −34.15, 95% CI [−42.26, −25.79]), and *OK* (Δ = −31.89, 95% CI [−37.25, −26.57]). Contrastingly, the distributions of *oui*, *mm‐hm / hein‐hein*, repetitions, and *ouais* were skewed toward horizontal transitions, as their “horizontal” ratios were estimated to increase by 37.81 percentage points for *oui* (95% CI [30.15, 45.31]), by 35.46 for *mm‐hm / hein‐hein* (95% CI [27.87, 42.94]), by 20.9 for repetitions (95% CI [14.07, 27.80]), and by 13.23 for *ouais* (95% CI [7.99, 18.43]). Lexical markers *oui*, *mm‐hm / hein‐hein*, repetitions, and *ouais* thus function as horizontal markers, while *voilà*, *exactement / exact*, and *OK* as vertical markers.

#### 3.3.3 Vertical and horizontal markers in Vietnamese

From *N* = 9602 extracted markers, we identified 10 linguistic forms in Vietnamese (see Table 2): *rồi* ^2^ (“all right,” 26.9%), *ô kê* (“okay,” 20.7%), *ừm* or *ừm‐hứm* (“mm, uh‐huh,” 12.6%), *ờ* or *ừ* (“yeah,” 12.4%), repetitions or restatements (12%), *đúng* or *đúng rồi* (“right,” 9.5%), *được* or *được rồi* (“right, okay,” 3%), *vâng*, *dạ*, or *dạ vâng* (“yes,” 1.8%), *chính xác* (“correct,” 0.6%), *chuẩn* (“exactly,” 0.5%). We further analyzed six most frequent linguistic forms *rồi*, *ô kê*, *ừm / ừm‐hứm*, *ờ* */* *ừ*, repetitions, and *đúng* *rồi* that represented 94.2% (*N* = 9046) of all extracted markers.

As stand‐alone tokens, Vietnamese participants deployed minimal forms *ừm / ừm‐hừm* (88.8%), response tokens *vâng*, *dạ*, or *dạ vâng* (79.8%), acknowledging *ô kê* (66%), and assessment *chính xác* (64.3%). As within‐turn markers, participant tended to deploy turn‐prefacing *chuẩn* (81.5%). Other forms of coordination markers and combinations of various forms were distributed somewhat equally between stand‐alone and within‐turn contexts (see S1, Section 4.2). In Section 5.2 of S1, we additionally provide the relative frequency distributions of each marker form as deployed by builders versus directors.

Model 4 detected transition‐specific biases in the proportional distributions of all six linguistic forms (Figure 4.2). Markers *ô kê*, *rồi*, and *đúng rồi* were mostly employed in vertical transitions. The model predicted a substantial decrease in “horizontal” ratios of *ô kê* (Δ= −46.61, 95% CI [−52.58, −40.49]), *rồi* (Δ= −38.84, 95% CI [−44.59, −33.09]), and *đúng* */* *đúng rồi* (Δ= −17.91, 95% CI [−26.04, −9.89]). In contrast, the average “horizontal” proportions of repetitions, *ừm / ừm‐hứm*, and *ờ* */* *ừ* increased by 41.13 percentage points for repetitions (95% CI [34.43, 47.69]), by 23.37 for *ừm / ừm‐hứm* (95% CI [16.28, 30.39]), and by 19.4 for *ờ / ừ* (95% CI [12.18, 26.43]). Given the predicted distributions of Vietnamese markers, repetitions, *ừm / ừm‐hứm*, and *ờ/ ừ* serve as horizontal markers, and *ô kê*, *rồi*, *đúng rồi* as vertical markers.

#### 3.3.4 Vertical and horizontal markers in Shipibo‐Konibo

We categorized *N* = 11,728 Shipibo‐Konibo coordination markers into seven linguistic forms (see Table 2 for their phonetic transcriptions): *aya* or *ya* (“yes, okay, all right,” 55.1%), *jejen* and its variations *ajan / jem / mm* (“mm, uh‐huh, mm‐hm,” 27.8%), repetitions or restatements (6.5%), *moa* ^3^ (“that's it,” 4.3%), *jaskara* (“like so,” 3.1%), *aita* (*ahí ta* “there it is,” 1.6%), and *other* (1.6%). Within the category *other* (*N* = 184), we identified low‐frequency markers such as markers of assessment *jakon* (“good,” *N* = 70), *perfecto* (“perfect,” *N* = 2), *exacto* (“correct,” *N* = 1), markers of acknowledgment *listo* (“okay, ready,” *N* = 81), *okey* (“okay,” *N* = 19), and *si* (“yes,” *N* = 4), and a change‐of‐state token *eri* (“oh,” *N* = 7). With model 5, we analyzed by‐transition distributions of five most frequent markers *aya / ya*, *jejen / ajan / jem / mm*, repetitions, *moa*, and *jaskara* (*N* = 11,353, 96.8%). Among these various forms, markers *listo* (77.3%), *moa* (77.2%), *jejen / ajan / jem / mm* (68.1%), and *jaska‐* (66.3%) appeared mainly as stand‐alone tokens, while *aita* (61.6%) and *jakon* (83.9%) were deployed within an extended turn. All other forms appeared in both turn positions with somewhat equal frequencies (see Section 4.3 of S1). By‐form frequency distributions of coordination markers between participant roles can be found in Section 5.3 of S1.

For all marker forms, model 5 predicted substantial differences in the proportional distributions between horizontal and vertical transitions (Figure 4.3). For horizontal transition contexts, we observed a decrease in proportional distributions of *moa* (Δ = −53.09, 95% CI [−59.95, −45.91]), *jaskara* (Δ = −21.8, 95% CI [−31.14, −12.47]), and *ya* (Δ = −11.80, 95% CI [−17.01, −6.59]). The average ratios of the remaining markers *jejen / ajan / jem / mm* and repetitions, on the contrary, were predicted to increase in the vicinity of horizontal transitions by 63.12 for repetitions (95% CI [57.46, 68.41]) and by 28.59 points for *jejen / ajan / jem / mm* (95% CI [23.20, 33.94]). The model thus estimated that markers *jejen / ajan / jem / mm* and repetitions are used as horizontal markers, while *moa*, *jaskara*, and *ya* as vertical markers.

### 3.4 Variation in proportional distributions of repetitions, minimal and lexicalized markers between horizontal and vertical transitions

Lastly, we tested whether coordination markers expressed as repetitions, minimal, and lexicalized words showed associations with specific transition contexts, and if these associations were consistent across languages.

#### 3.4.1 Model specifications

For models 6.1 and 6.2, we regrouped all extracted coordination markers into three categories according to their linguistic forms (Table 3). Next, we aggregated all extracted markers from three languages into a single dataset and assigned each marker a corresponding marker category (lexicalized, minimal, or repetitions). For each marker category (e.g., minimal), we extracted cumulative marker counts per transition context (minimal_horizontal_ and minimal_vertical_). Each data entry (*N* = 1311) contained participant ID, marker category label, marker counts per transition context (π), and total category counts in both transitions ($n_{category\;total}$). Model 6.1 was fitted as a beta binomial regression that inferred the probability distributions of marker categories between two transition contexts irrespective of language:

(6.1) $$\begin{array}{cc} \mathrm{marker}\;\mathrm{category}\;\mathrm{per}\;{\mathrm{transition}}_{i} & ∼\mathrm{Beta}\;\mathrm{Binomial}(n_{cat.\;total},\pi_{i},\phi )\\ logit\left(\pi_{i}\right)\;|\;\mathrm{trials}\;\left(n_{\mathrm{cat}.\;\mathrm{total}}\right) & =\beta_{1}{\mathrm{category}}_{i}+\beta_{2}{\mathrm{transition}}_{i}+\\ & +\beta_{3}\left({\mathrm{category}}_{i}\times {\mathrm{transition}}_{i}\right)+\\ & +(1\;|\;\mathrm{participant}\;\mathrm{ID}) \end{array}$$

Model 6.2 measured whether the trends estimated by model 6.1 held within each target language. We thus included language (Swiss, Vietnamese, or Shipibo‐Konibo) into a three‐way interaction with marker category and transition type:

(6.2) $$\begin{array}{cc} \mathrm{logit}\left(\pi_{i}\right)\;|\;\mathrm{trials}\;\left(n_{\mathrm{cat}.\;\mathrm{total}}\right) & =\beta_{1}{\mathrm{category}}_{i}+\beta_{2}{\mathrm{transition}}_{i}+\beta_{3}{\mathrm{language}}_{i}+\\ & +\beta_{4}\left({\mathrm{category}}_{i}\times {\mathrm{transition}}_{i}\right)+\beta_{5}({\mathrm{category}}_{i}\times \\ & \times {\mathrm{language}}_{i})+\beta_{6}\left({\mathrm{transition}}_{i}\times {\mathrm{language}}_{i}\right)+\\ & +(1\;|\;\mathrm{participant}\;\mathrm{ID}) \end{array}$$

As weakly informed priors, we set $\beta_{1},\beta_{2},\beta_{3}∼$ Normal (0,1).

**Table 3:** Categories of coordination markers across three target languages

	Target languages		
Marker category	Swiss French	Vietnamese	Shipibo‐Konibo
lexicalized	*ouais, OK, oui, voilà, exactement, parfait, d'accord, top, tip top, magnifique, super, effectivement, tac, correct*,	*rồi, ô kê, ờ, ừ, đúng, đúng rồi, được rồi, vâng, dạ, chính xác, chuẩn*	*aya, ya, moa, jaskara, aita, exacto, jakon, perfecto, listo, okey, si*
minimal	*mm‐hm, hein‐hein, mm*	*ừm, ừm‐hứm*	*jejen, ajan, jem, mm, eri*
repetition	repetitions, restatements, or summary statements produced as response markers to acknowledge the previous utterance(s)		

#### 3.4.2 Effects of marker category

Model 6.1 revealed that transition contexts substantially influenced the choice of marker category (Figure 5a,b). Consistent with our predictions, more lexicalized forms of markers were least likely to appear in horizontal transitions (Δ =−14.09, 95% CI [−16.87, −11.28]). However, for minimal markers, the model predicted a 29.10 point increase in horizontal contexts (95% CI [25.55, 32.67]). Repetitions showed the strongest association with horizontal transitions, with an average predicted increase of 42.98 points (95% CI [39.31, 46.55]).

Model 6.2 tested whether these distributions were observed in each target language. Model output revealed that these trends were rather stable in all languages, being stronger in Vietnamese and Shipibo‐Konibo than in Swiss French (Figure 5c,d). In Vietnamese, the predicted ratios in horizontal transitions increased for repetitions by 43.72 percentage points (95% CI [38.11, 49.14]) and for minimal markers by 24.19 points (95% CI [17.58, 30.48]), while decreased by 23.42 points for more lexicalized markers (95% CI [−27.84, −18.94]). In Shipibo‐Konibo, horizontal transitions predicted a substantial increase in the use of repetitions (Δ = 63.79, 95% CI [58.36, 68.84]) and minimal markers (Δ = 29.17, 95% CI [24.37, 34.09]), and a decrease in the use of lexicalized forms (Δ = −13.55, 95% CI [−18.32, −9.02]). In Swiss French, horizontal contexts predicted a larger increase for minimal markers (Δ = 36.17, 95% CI [28.85, 43.71]) than for repetitions (Δ = 21.62, 95% CI [15.13, 27.71]). Consistent with the distributions in other languages, we observed the negative effect of horizontal contexts on the use of more lexicalized forms. However, the decrease was estimated to be slightly weaker than in Vietnamese and Shipibo‐Konibo (Δ = −5.18, 95% CI [−9.77, −0.68], *pd* = 98.8%).

In all language subsets, minimal markers and repetitions were predicted to appear rather in horizontal transitions, while lexicalized markers in vertical transitions. In all target languages, participants showed a strong preference to choosing minimal markers and repetitions in horizontal transition contexts. The association between lexicalized markers and vertical transitions, however, was stronger in Vietnamese and Shipibo‐Konibo than in Swiss French.

## 4 Discussion

In this paper, we explored how speakers from three linguistically and culturally unrelated populations (Switzerland, Vietnam, and the Peruvian Amazon) use conversation markers like *uh‐huh*, *yeah*, *okay* as specialized coordination markers to navigate the progress in the course of task‐oriented joint activities. Goal‐directed activities (here, *tasks*) unfold in hierarchies of *sub‐tasks* and smaller *sub‐subtasks* (Zacks & Tversky, 2001). Coordination of such hierarchies in joint activities implies that participants need to agree whether they are continuing an ongoing sub‐task, that is, transitioning *horizontally*, or switching to the next sub‐task, that is, transitioning *vertically* (Bangerter & Clark, 2003). As language serves to regulate joint activities (Clark, 1996), its use should reflect the distinction between horizontal and vertical transitions. We empirically tested this assumption in three maximally diverse (Stoll & Bickel, 2013) languages with the same joint activity task, in which dyads of directors and builders jointly built LEGO models (Clark & Krych, 2004).

First, we tested whether the production of coordination markers was influenced by the language, sex, and familiarity of the participants. We found strong evidence that speakers of all target languages frequently employed coordination markers, albeit with varying production rates. Swiss French speakers showed the highest rates of coordination marker production, followed by Vietnamese, and lastly Shipibo‐Konibo participants. Our results align with previously reported findings on differences in the amount of grounding feedback across cultures. The amount of coordination markers produced by participants might vary due to a number of factors, including differences in available feedback‐eliciting cues in target languages (Maynard, 1986), or whether participants from certain cultures rely more on nonverbal strategies of signaling attention and understanding (Stubbe, 1998), or even culture‐specific politeness norms regulating the acceptable amount of grounding feedback, especially given that it is often produced in overlap with the current speaker (Stubbe, 1998). Lastly, in this study, we focused only on lexical coordination markers, disregarding other potential grounding devices such as affirmative question words and grammatical markers of agreement. Quantifying the role of agreement affixes as transition markers in joint activities would be especially relevant for languages with polysynthetic tendencies such as Shipibo‐Konibo.

We also found only weak evidence that coordination marker production varies as a function of sex and familiarity of the participants. Contrary to the previous findings suggesting that women produce more listener feedback (Bilous & Krauss, 1988; Hirschman, 1994; Mulac & Bradac, 2012; Roger & Schumacher, 1983; Roger & Nesshoever, 1987), we did not find reliable evidence that participants' sex affected the production rates of coordination markers. Instead, we observed marginal increase of marker production rates in male‐male and mixed‐sex dyads, yet not in all target languages. With regard to familiarity, there is weak evidence that dyads of strangers and slightly familiar participants produce more verbal coordination markers than dyads of friends and family members. While this finding is consistent with the idea that less familiar participants put more communicative effort into maintaining the common ground in a dialogue (Jucker & Smith, 1998), this effect was detected only in Shipibo‐Konibo participants. One possible explanation could be rooted in the differences in day‐to‐day environments of interactions at the sites of our study. Swiss and Vietnamese participants came from industrialized, densely populated urban centers, where they might frequently engage in joint activities with strangers. In Shipibo‐Konibo communities, on the other hand, most villagers share extended kinship connections within the community and even with neighboring villages (De Carvalho Rodrigues Lopes, 2022). As their daily social encounters revolve mainly around their relatives and friends, they might display so‐called “closeness‐communication bias,” thereby overextending the common ground to irrelevant, novel communicative contexts (e.g., the LEGO‐building task) and, therefore, putting less effort to maintain communicative alignment with friends and family compared to strangers (Nickerson, 1999; Van Der Wege, Jacobsen, Magats, Mansour, & Park, 2021). It is possible that the effects of familiarity on coordination marker production might be less pronounced in Shipibo‐Konibo speakers living in urban centers, for example, in Pucallpa, Lima, or Ica. In summary, we found that the use of verbal coordination markers is an important component of joint action coordination, regardless of the sex, language, or familiarity of the participants. If present, the effects of sex and familiarity are rather culture‐specific than universal.

Despite the differences in the production rates of coordination markers, participants in all languages attuned their use of coordination markers to the manipulations in the visibility (visible vs. hidden conditions) and their respective roles in model building (director vs. builder). The majority of coordination markers were produced in the hidden condition when participants coordinated model construction solely through vocal communication. However, the production of verbal coordination markers in the visible condition decreased greatly because (1) participants could engage in nonverbal coordination by establishing mutual gaze, nodding, or gesturing, and (2) there was no need to acknowledge completion of the construction steps. Our findings support the effects of copresence and visibility on the reduction of grounding costs in the visible condition (Clark & Schaefer, 1987; Clark & Brennan, 1991). We also observed the language‐invariant trend of the division of collaborative contributions between experimental conditions. In the hidden condition, the directors could not monitor the actions of the builders and they had to rely on cues provided by the builders to track the construction progress. Builders thus produced more coordination markers than directors, dutifully using them to ground directors' instructions and signal the completion of construction steps. In the visible condition, there was no need for the builders to ground the directors' instructions verbally. Instead, they could display blocks or block positions, trusting directors to either ratify their actions with coordination markers or promptly correct them. This division of collaborative contributions reflects the corepresentation between participants (e.g., Sebanz, Knoblich, & Prinz, 2003; Sebanz & Knoblich, 2009; Vesper, Butterfill, Knoblich, & Sebanz, 2010) that allows them to anticipate certain contributions from the other and adjust their own contributions (or lack thereof) accordingly.

Next, we documented the repertoires of lexical coordination markers in each target language and explored language‐specific and language‐invariant forms of horizontal versus vertical markers. The analysis of how speakers deploy coordination markers between horizontal and vertical transitions suggests that in each language, these words exhibit strong associations with either transition type. It implies that speakers strategically choose linguistic forms of coordination markers to provide a conventionalized distinction between horizontal and vertical transition contexts. In Swiss French, horizontal transitions are systematically marked with *oui, ouais, mm‐hm, hein‐hein, mm*, and repetitions, while vertical transitions with *voilà, exactement*, and *okay*. In Vietnamese, markers *ừm, ừm‐hứm, ờ, ừ*, and repetitions are used as horizontal markers and *ô kê, rồi, đúng rồi* as vertical markers. In Shipibo‐Konibo, words *jejen / ajan, jem, mm*, and repetitions function as horizontal markers and *moa*, *jaskara*, and *ya* as vertical markers.

Our study quantifies and assesses the use of repetitions as potential coordination markers, offering a novel contribution to the limited body of research on the cross‐linguistic use of repetition for communicative grounding (see Zellers, 2021). Furthermore, our study provides additional evidence on the diffusion of *okay* as coordination marker in languages beyond English (Bangerter et al., 2024; Betz et al., 2021). Not only was *okay* the second most frequent marker in Swiss French and Vietnamese, it was also consistently used near vertical transitions, just like in English (Bangerter & Clark, 2003). In Shipibo‐Konibo, the most frequent marker was vertical *ya* from Andean Spanish, where it is also used in vertical contexts, such as introducing new topics or marking digression boundaries (Fuentes‐Rodríguez, Placencia, & Palma‐Fahey, 2016; García Tesoro, 2022; Shappeck, 2013). Our findings suggest that languages, likely through extended language contact, might adopt foreign coordination markers while preserving their functional use.

Further analysis supported our predictions that the distribution of linguistic forms between transition‐specific contexts is not arbitrary. Even across maximally diverse languages, speakers favor the production of minimal markers (*uh‐huh, mm, mm‐hm*) and repetitions in horizontal transitions and more lexicalized marker forms (*yeah, okay, right*) in vertical transitions. This finding suggests that vertical and horizontal markers might be subjected to different sets of selective pressures. Given that the primary interaction environment of horizontal transitions are ongoing sub‐tasks of joint activities, participants should opt for mitigating the dual‐tasking load of carrying out the activity and providing verbal coordination. In this case, preferred coordination markers would be nondisruptive markers of acknowledgment that do not introduce new content and, therefore, do not shift the point of attention from the task at hand. In addition, they should be easier to plan and produce. Minimal markers such as *mm, mm‐hm*, and repetitions have been previously shown to fulfill these functions (Bartolozzi et al., 2021; Dingemanse et al., 2022; Knudsen et al., 2020; Svennevig, 2004). Vertical transitions, on the other hand, occur between sub‐tasks of an activity and should, therefore, be associated with reduced dual‐tasking load. They should also effectively prompt the shift of attention to the next sub‐task. If this is the case, participants should be able to plan and produce the markers that bear more meaning and show a higher degree of incipient speakership (Drummond & Hopper, 1993; Jefferson, 1984), as it warrants more attention from their interaction partner. Here, we provide indirect evidence suggesting the differences in dual‐tasking loads between the two generic transitions in the joint activity hierarchy. Further neurolinguistic studies are needed to assess the difference in processing loads between horizontal and vertical transitions, as well as estimate the planning and production efforts of minimal markers and repetitions versus more lexicalized markers.

Our study has a number of limitations. First, we focused only on verbal coordination markers. As we have shown in our study, the use of verbal markers decreases when participants can monitor each other's faces and actions, suggesting that nonverbal cues play an important role in joint action coordination. Visual cues such as head nods and (to a lesser extent) head tilts and smiles serve as the most frequent devices for maintaining discourse continuity not only among BSL signers but also in English speakers (Lutzenberger, De Wael, Omardeen, & Dingemanse, 2024). Besides head nods and smiles, there is a number of other nonverbal behaviors that could be used to regulate joint activities in humans and nonhuman animals, for example, gaze, body movements, gestures, facial expressions, body orientation, and some nonverbal vocalizations such as laughter (for a review, Heesen et al., 2017). In addition, the production and recognition of nonverbal behavioral cues play an important role in supporting joint action coordination in young children (Brownell, Ramani, & Zerwas, 2006; Siposova, Tomasello, & Carpenter, 2018; Warneken, Chen, & Tomasello, 2006).

Next, our findings indicate that all forms of verbal coordination markers might appear in both transition contexts, but with different probabilities. Lexical cues might, therefore, not always be sufficient to infer whether interaction partners wish to proceed horizontally or vertically. In fact, there is evidence that acoustic cues differentiate horizontal markers from vertical markers (Morozova, You, Stoll, & Bangerter, 2024). Multiple articles have previously reported context‐dependent changes in prosody, duration, and intensity of words such as *uh‐huh, yeah, okay* (Beach, 2020; Benus, Gravano, & Hirschberg, 2007; Couper‐Kuhlen, 2021; Hockey, 1993; Jurafsky et al., 1998; Shriberg et al., 1998; Truong, Poppe, & Heylen, 2010). It is then up to future research to explore which acoustic features distinguish horizontal markers from vertical markers, and if such features are language‐specific or potentially universal.

## 5 Conclusion

We have shown that participants from linguistically and culturally diverse populations use a special class of linguistic conventions, here *coordination markers*, to navigate joint activities. Humans conceptualize goal‐directed activities as hierarchies of activity sub‐components and employ coordination markers to distinguish the continuation of ongoing sub‐tasks (with designated horizontal markers) from switching between sub‐tasks (with designated vertical markers). Despite a large variety of coordination marker forms and their linguistic realizations across languages (e.g., *uh‐huh, mm, yeah, right, okay, perfect, that's it, there you go*), their function and transition‐specific properties of their linguistic forms allude to potentially language‐invariant patterns of their use in joint action coordination. Specifically, minimal markers and repetitions are preferentially deployed in horizontal transitions, while more lexicalized markers in vertical transitions, even in unrelated languages with maximally diverse typological features. Our study contributes to the ongoing dialogue on how conversational infrastructure (in our case, coordinating transitions in joint activities) might selectively shape linguistic conventions in the process of convergent language evolution.

## Supporting information

Data S1

## Notes

1 Anonymized data and analyses are available at OSF.io.

2 We separated affirmative and acknowledging *rồi* used as a coordination marker from tense marker *rồi*.

3 We separated affirmative and acknowledging *ya* and *moa* used as coordination markers from tense markers.
