# Supplementary material for: Language‐Invariant Strategies of Navigating Transitions in Joint Activities: Forms and Functions of Coordination Markers
Source: Cogn Sci. 2025 Nov 26;49(11):e70133. doi: 10.1111/cogs.70133 (PMC12658410; doi:10.1111/cogs.70133)
Supplement: Supplementary file 1 — Data S1 [file COGS-49-e70133-s001.pdf]

Supporting information

Natalia Morozova[1], Sabine Stoll[1], and Adrian Bangerter[2]

[1]Institute for the Interdisciplinary Study of Language Evolution (ISLE Institute), University of Zurich
[2]Institute of Work and Organizational Psychology (IPTO), University of Neuchâtel

Contents

1 Annotation conventions **3**
1.1 Model . . . 3
1.2 Condition . . . 3
1.3 Phase . . . 3
1.3.1 Entry . . . 3
1.3.2 Main body . . . 4
1.3.3 Exit . . . 5
1.4 Sub-phase . . . 5
1.4.1 Block PL . . . 5
1.4.2 Block ID . . . 6
1.4.3 Block CH . . . 6
1.5 Blocks . . . 7
1.6 Transitions . . . 7
1.6.1 Vertical transitions . . . 8
1.6.2 Horizontal transitions . . . 8
1.7 Question-answer sequences . . . 9

2 Prototype models **11**
2.1 Figure 1 . . . 11
2.2 Figure 2 . . . 11
2.3 Figure 3 . . . 11
2.4 Figure 4 . . . 12
2.5 Figure 5 . . . 12
2.6 Figure 6 . . . 12
2.7 Figure 7 . . . 13
2.8 Figure 8 . . . 13
2.9 Figure 9 . . . 13
2.10 Figure 10 . . . 14
2.11 Figure 11 . . . 14

3 COVID-19 safety measures **14**
3.1 Research team 14
3.2 Changes in the experimental setup 15
3.3 Changes in the procedure 16

4 Distribution of marker forms between stand-alone and within-turn contexts **17**
4.1 Swiss French 18
4.2 Vietnamese 18
4.3 Shipibo-Konibo 19
4.4 Distribution of horizontal and vertical markers between stand-alone and within-turn contexts 19

5 Distribution of marker forms between participant roles (directors vs. builders) **20**
5.1 Swiss French 20
5.2 Vietnamese 21
5.3 Shipibo-Konibo 21

1 Annotation conventions

Participants' speech was manually segmented, transcribed, translated, and annotated in ELAN `v.6.3`. Annotators received .mp4 video recordings, .wav audio files recorded by clip-on microphones, and .eaf files with pre-segmented participant speech. Participants' speech segments were annotated at seven levels (`model`, `condition`, `phase`, `sub-phase`, `block`, `transition`, `q&a`), and each level had corresponding tiers for director's (marked with `@d`) and builder's speech (marked with `@b`). Annotators worked in Options » Transcription mode.

Annotation of each session started from Model 1. The trial began when the prototype model was placed in the box in front of director. The trial ended when (a) participants announced the end of construction to the experimenter or (b) when director took the model out of the box shortly before announcing the end of construction. Annotators worked only with speech segments produced during trials by participants and addressed to the other participant. Experimenter's speech and participants' speech addressed to the experimenter (noted in the Comments by transcribers) were ignored.

1.1 Model

Model numbers were annotated in ELAN tier column `model` and corresponding tiers `model@d` and `model@b`. Models were administered in the same order and were tagged in order of their appearence as `mod1`, `mod2`, `mod3`, `mod4`, `mod5`, `mod6`, `mod7`, `mod8`, `mod9`, or `mod10`.

1.2 Condition

Conditions were annotated in ELAN tier column `condition` and the corresponding tiers `condition@d` and `condition@b`. Participants built five consecutive models in either condition. The order of conditions was counterbalanced among dyads. Annotators were asked to refer to the .mp4 video materials to check if participants were separated with a cloth (`hidden` condition) or could see each other through the safety screen (`visible` condition).

1.3 Phase

Phases were annotated in ELAN tier column `phase` and the corresponding tiers `phase@d` and `phase@b`. Each trial could contain up to three phases: `Entry`, `Main body`, and `Exit`. Not every trial contained Entry and Exit; as participants moved through the trials, they were less likely to acknowledge the beginning and the end of model construction, especially in visible condition.

1.3.1 Entry

`Entry` phase included all utterances that preceded the model construction process, including:

1. attention-drawing utterances, e.g.

> `director:` *Hey <participant name>!*
> `builder:` *Yeah?*

2. explicit indications of the trial start, e.g.

> `director`: *Okay, let's start*
> `builder`: *Yeah... Describe from the bottom up*

or

> `director`: *This is the eightth model, my friend! Ta-da!*
> `builder`: *I know you too well* <chuckles>
> `director`: <chuckles> *Next...*

3. negotiations of the trial start, usually indicated by opening questions, e.g. *Are you ready?* or *Can we start?* The negotiation might continue if the other participants is not ready, e.g. *Wait wait, let me put it away. Okay, we can start.*

4. extended reactions (two or more turns) to the prototype model, e.g.

> `director`: *Wow!*
> `builder`: <laughs>
> `director`: <laughs>
> `builder`: *Stay calm*

5. irrelevant talk, e.g.

> `builder`: *Should we play LEGO later? Then I can describe and you build*
> `director`: <laughs> *No*
> `builder`: *Come on, it could be fun too, right?*

`Entry` phase finished as soon as participants started discussing the model (*Now let me count*) or began assembling it (*First, take a green block*).

1.3.2 Main body

All speech segments related to the completion of the experimental task were annotated as part of the main phase, or `Main body`. Some directors opened a trial by sharing their impressions of a model (e.g. *It looks kinda like an airplane*), counting the amount of blocks (*Okay, there will be six pieces in total*), or indicating that they were preparing to provide instructions (*Let me count first*). Unlike `Entry` phase segments that were directed towards negotiating the trial start, `Main body` segments were directly related to the task even if participants did not mention any blocks yet.

Often trials began with coordination markers *okay, all right* or fillers *er, um*. Since these words do not explicitly mark the trial start, they were annotated as part of `Main body`. Similarly, trials often ended with coordination markers or fillers, e.g.

> `director`: *Just put it in the middle*
> `builder`: *Er... Okay*
> `director`: *Okay*
> `director`: <to the experimenter> *Okay, we are done*

1.3.3 Exit

`Exit` phase included all speech segments that followed the end of model construction, including:

1. attention-drawing utterances, e.g.

> `director:` *Hey <participant name>, are you done?*
> `builder:` *I think so*
> `director:` *Okay*

2. explicit indications of the trial end, e.g.

> `director:` *Yeah, okay, that's done*
> `builder:` *Done, but it's falling apart*
> `director:` <laughs> *Okay*

3. negotiations of the trial end, usually indicated by opening questions, e.g. *Are you done? Is it over?* or *Should we compare?* The negotiation might continue if the other participant was not ready, e.g. *Wait a second*, or expressed uncertainty, e.g. *That's it? Are you sure? What if the models are different, I am scared.*

4. irrelevant talk, e.g.

> `director:` *Done*
> `builder:` *Is that all?*
> `director:` *Yeah*
> `builder:` *Yeah*
> `director:` *It looks a bit like a guy jumping down, as if he wants to jump off the rock*
> `builder:` <laughs> *Okay, nice imagination*
> `director:` <laughs>

or

> `builder:` *I am gonna kill you if you instructed me wrong*
> `director:` *Oh my gosh, this builder is so dramatic*

`Entry` and `Exit` phases were further annotated only for `transition`, `q&a` and `coordination marker`. Only `Main body` phase included additional annotations for `sub-phase` and `block`.

1.4 Sub-phase

Sub-phases were annotated in ELAN tier column `sub-phase`, in the corresponding tiers `sub-phase@d` and `sub-phase@b`. Trials could include up to three sub-phases: `Block ID`, `Block PL`, and `Block CH`. However, additional coordination routines `Block ID` and `Block CH` were not present in every trial.

1.4.1 Block PL

Block Placement sub-phase, or `Block PL`, implied the identification and immediate placement of model blocks, e.g.

> `director:` *Now you take a long green two-by-eight*
> `builder:` *Okay, I see it*
> `director:` *Put it horizontally. Next, find a round red block*
> `builder:` *A round red... Ah yeah, here it is*
> `director:` *There, you put it on the left side of the green block*
> `builder:` *Okay, next*

1.4.2 Block ID

Occasionally, participants decided to find all model blocks before compiling them into a model. We called such pre-constuction block identification routine `Block ID` sub-phase. Unlike the main construction routine `Block PL`, `Block ID` did not include immidiate placement instructions after block descriptions. Directors could choose to describe all six blocks or smaller block sub-sets. In some cases, directors simply described the model's colour palette, e.g. *So it has several shades of blue, some purple and pink, and one block is transparent.*

Participants rarely explicitly acknowledged the beginning of `Block ID` sub-phase, e.g. *So here are the materials you will need.* However, the transition between `Block ID` and `Block PL` sub-phases was often explicitly acknoweldged, e.g. *That's all, now you put them together* or *Let's start*, or *Now let's assemble.*

1.4.3 Block CH

This additional construction routine refers to the situations when participants decided to check the configuration of the whole model or some specific blocks. Here we call this sub-phase Block check, or `Block CH`. This sub-phase could be encountered when:

1. directors asked builders to re-describe what they have built (usually in hidden condition)
2. participants realised that some blocks must have been placed incorrectly, which prevented them from placing the current block
3. in visible condition, directors asked builders to raise and rotate the model (to assess its configuration from different angles).

If participants returned to previously placed blocks to reposition them, annotators were instructed to tag such segments as `Block CH` only if participants fixed **more than one block.** In the following example, participants returned to one previously placed block and therefore stay within Block PL sub-phase:

Entry » B1 » B2 » B3 » B4 » B5 » B4 » B5 » B6 » Exit

However, here participants discussed and/or re-positioned multiple blocks, i.e. they went through mid-trial Block CH sub-phase:

Entry » B1 » B2 » B3 » B4 » B5 » B4 » B3 » B2 » B3 » B4 » B5 » B6 » Exit

Often participants explicitly opened `Block CH` sub-phase, e.g. *Okay, wait let me start again* or *Should I describe it again and you check?* If present, Block ID and Block CH sub-phases were further annotated for `block` and `q&a` but **excluded from** `transition` **annotation and** `coordination marker` **extraction due to uncertainties in their hierarchical organisation.**

1.5 Blocks

Blocks were annotated in `block` tier column, in the corresponding tiers `block@d` and `block@b`. Each model was designed to include six blocks, i.e. `b1, b2, b3, b4, b5, b6`. However, participants could have forgotten to include one block or accidentally used more blocks than in the original design; in such cases, annotators used more or less labels. If participants placed two blocks in parallel, annotations included all respective block labels. If participants talked about the whole model, annotators could tag such segments as `model`, e.g.

> `director:` *Okay, it has one two three four, it has four floors in total, okay?*
> `builder:` *Uh-huh*

Block labels were assigned in order of their appearance in a trial and remained the same throughout all sub-phases. Change of blocks occurred when participants agreed to move to another block. **Change of blocks did not occur** in situations when:

1. participants simply mentioned the referential position of other blocks to determine where they should have placed the current block, e.g.

> `director:` *It should be placed at the um the edge, next to that brown cylinder*
> `director:` *And then its slope should face the yellow block*
> `builder:` *Uh-huh*

2. director kept providing instructions even if builder asked to move to the next block, e.g.

> `director:` *It will fit perfectly, that's right*
> `builder:` *But it's not... okay*
> `director:` *And its sides*
> `builder:` *Done, next*
> `director:` *Its sides are symmetrical with this orange block*
> `builder:` *Symmetrical, okay*

3. participants simply produced coordination markers without any instructions. Coordination marker exchange at the end of block sub-tasks served as an implicit negotiation to move to the next sub-task (and thus was treated as part of the current block sub-task), e.g.

> `director:` *Now put it horizontally*
> `builder:` *Okay, horizontally, the row with eight studs is now... er parallel to me*
> `director:` *Yeah*
> `director:` *All right*
> `builder:` *Okay*

1.6 Transitions

Transitions were annotated in `transition` tier column, in the corresponding tiers `trans@d` and `trans@b`. Transitions marked changes in the hierarchical structure of a joint activity. Transitions **between** sub-components of the joint task were tagged as `vertical` and **within** block sub-tasks as `horizontal`.

1.6.1 Vertical transitions

Vertical transitions occurred between phases (`Entry` to `Main body`, `Main body` to `Exit`), between sub-phases (switches from `Block ID` to `Block PL`, from `Block PL` to `Block CH`), and between block sub-tasks (e.g. from `B1` to `B2`, from `B2` to `B3`, etc.) within the main construction routine (`Block PL` sub-phase).

Vertical transitions between blocks in `Block ID` and `Block CH` sub-phases **were not annotated.** Annotators only marked vertical transitions **between these additional sub-phases and the main construction routine** in `Block PL` by tagging the first and last speech segments produced within `Block ID` and `Block CH`, e.g.:

> `vertical` `director:` *Okay, model nine, let's do our best*
> `vertical` `builder:` *Okay, let's go*
> `vertical` `director:` *I will start with describing all the blocks you need, okay?*
> `stop transition annotations for Block ID`

`<participants go through Block ID and switch to Block PL>`

> `no transition` `director:` *And then we will need a transparent yellow two-by-one. That's all*
> `vertical` `builder:` *Okay*
> `vertical` `director:` *Now let's assemble*
> `vertical` `builder:` *Uh-huh*

Between block sub-tasks, vertical transition sequences started with the last instruction for the current block (e.g. B2) and ended with the first instruction for the next block (e.g. B3).

> `B2 horizontal director:` *You should have four dots empty in the first row, do you see that?*
> `B2 horizontal builder:` *Ah yes, in the first row, okay. So where do I put it?*
> `B2 vertical director:` *Yeah that's right, you put it in the first row, right in the middle*
> `B2 vertical builder:` *Okay, it fits now, continue*
> `B3 vertical director:` *The next one is transparent, only two studs*
> `B3 horizontal builder:` *Which colour?*

Vertical sequence borders were defined by instructions that contained some information regarding a block's identity or position. Segments containing just coordination markers, i.e. words that helped participants to agree on moving to the next block, were included in vertical transition sequences, e.g.:

> `B1 vertical director:` *Next is a long red piece*
> `B1 horizontal builder:` *Okay*
> `B1 horizontal director:` *Its length is eight, the width is two*
> `B1 vertical director:` *The long bar*
> `B1 vertical builder:` *Okay*
> `B1 vertical director:` *Done?*
> `B1 vertical builder:` *Okay*
> `B2 vertical director:` *There is a green piece*

1.6.2 Horizontal transitions

Horizontal transitions occurred within block sub-tasks, i.e. within the opening and closing instruction borders, e.g.

B1 vertical director: *A green piece*
B1 horizontal builder: *The longest?*
B1 horizontal director: *Eight-dots long*
B1 horizontal director: *Sixteen dots total*
B1 horizontal builder: *Okay*
B1 horizontal director: *Two dots width, eight dots length*
B1 horizontal builder: *Okay*
B1 vertical director: *Turn it horizontally*
B1 vertical builder: *Okay*
B2 vertical director: *A... brown*
B2 horizontal director: *A brown piece, eight dots*

In visible condition, some block sub-tasks did not have any horizontal transitions. Since directors could monitor builders' actions, directors' instructions became shorter and more precise, and builders did not have to ground the instructions verbally, e.g.

B1 vertical director: *Now find a thin grey block, probably the biggest one you have there*
B1 vertical director: *And place it horizontally*
B1 vertical director: *That's right*
B2 vertical director: *Okay, find me a yellow two-by-four*

The majority of horizontal transitions occurred in `Main body` phase; however, `Entry` and `Exit` phases could contain horizontal transitions if they were significantly prolonged (e.g. five segments or longer, excluding laughter and other non-verbal vocalisations):

Entry vertical builder: *Hey <participant name>, did you know that I loved playing with LEGO as a kid?*
Entry horizontal director: *Huh?*
Entry horizontal builder: *I very much liked playing with LEGO before*
Entry horizontal director: *Uh, me too*
Entry vertical builder: *Uh-huh, but unfortunately my family don't give me money to buy LEGO now, tsk*
Main body vertical director: *This one also has six blocks*

1.7 Question-answer sequences

Questions and answers were annotated in `q&a` column, in the corresponding tiers `q&a@d` and `q&a@b`. Questions were annotated with `q` and answers (if provided) with `a`. Since not all interrogative utterances contained linguistic question markers, transcribers and annotators were asked to rely on their intuition and prosodic cues to identify interrogative speech segments.

Question-answer sequences did not always contain answers, e.g.

director: *Now find me a flat blue block*
q builder: *How many studs does it have?*
q director: *Did you find it? The biggest one*
builder: *Okay, the biggest. Continue*

or could be extended by other question-answer sequences, e.g.

> `director:` *Next is a red one*
> `q1 builder:` *How many studs does it have?*
> `q2 director:` *What do you mean?*
> `a2 builder:` *Count the number of dots for me*
> `a1 director:` *Ah, there are eight dots in total*

or could be extended over several speech segments interrupted by long pauses, e.g.

> `q builder:` *How many studs does it have?*
> `a director:` *One two three four...*
> `a director:` *Twenty five, twenty six...*
> `a director:` *Forty two studs in total*

Since answer-tagged segments contained agreement tokens (e.g. *yeah, yes, right*) that resemble **coordination markers**, such segments were excluded from coordination marker extraction, e.g.

> `q director:` *Did you find it?*
> `a director:` *Yeah, the biggest block*
> `director:` ***Right, okay***
> `q director:` *Next, do you see a yellow two-by-four?*

Participants could use coordination markers like *okay?*, *all right?*, *yeah?* as interrogative words to elicit acknowledgment from the other participant. In such cases, these words served to maintain alignment, not elicit agreement, and thus were extracted as coordination markers, e.g.

> `q director:` *And then you place this block in the middle,* ***okay****?*
> `a builder:` *Uh-huh, done*

If these interrogative words successfully elicited identical markers, they were extracted as coordination markers, e.g.

> `q director:` *And then you place this block in the middle,* ***okay****?*
> `a builder:` ***Okay****, done*

Other types of tokens appearing in answer-tagged segments were considered agreement tokens and thus excluded from further analyses.

2 Prototype models

2.1 Figure 1

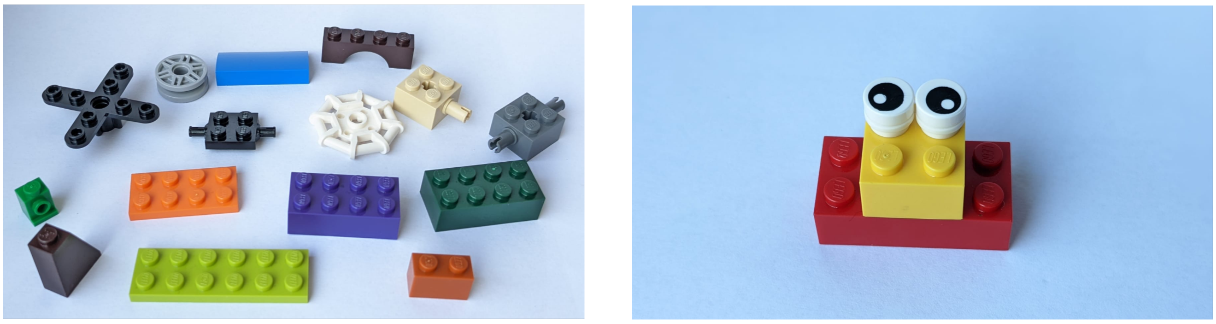

(a) (b)

Figure 1: Distracting pieces (a) and the test model (b).

2.2 Figure 2

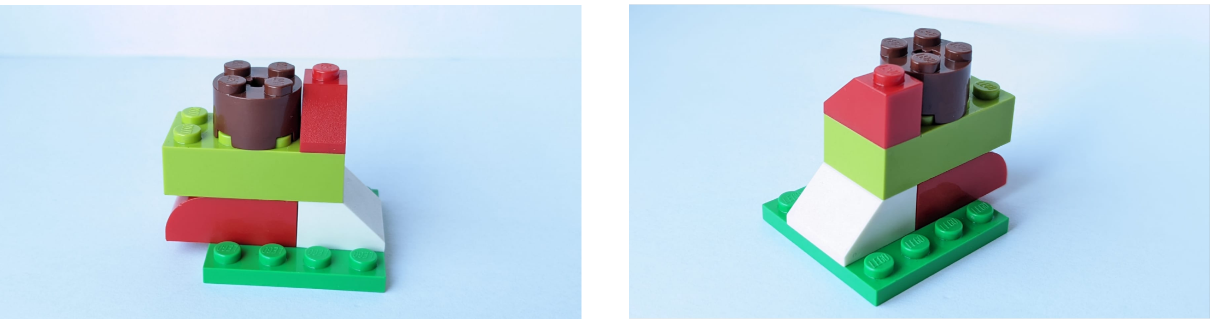

(a) (b)

Figure 2: Prototype Model 1 administered in Trial 1.

2.3 Figure 3

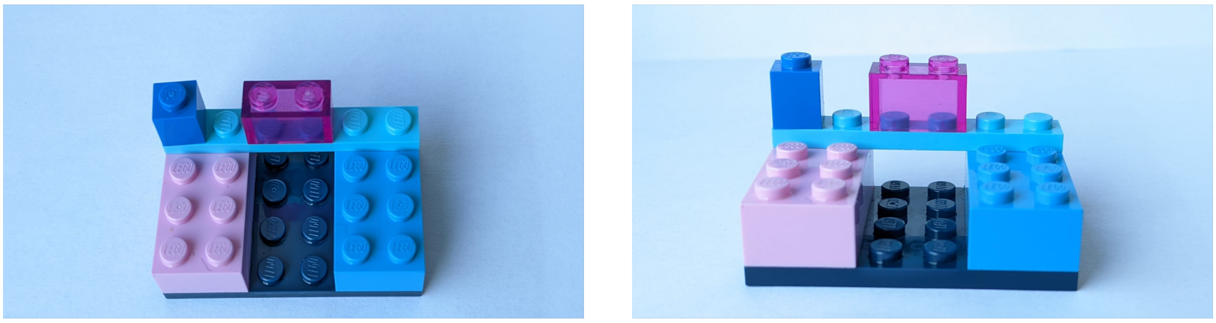

(a) (b)

Figure 3: Prototype Model 2 administered in Trial 2.

2.4 Figure 4

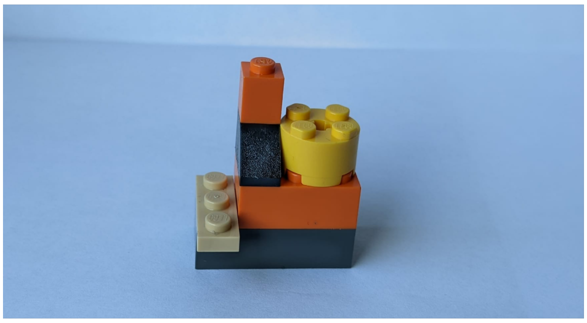

(a)

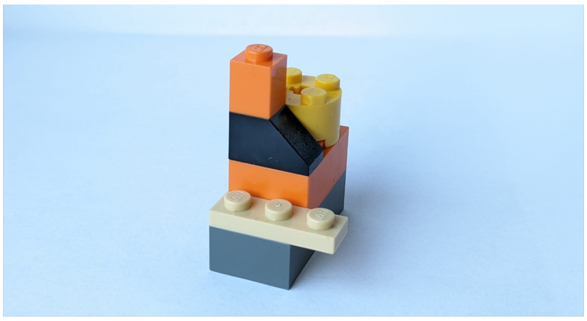

(b)

Figure 4: Prototype Model 3 administered in Trial 3.

2.5 Figure 5

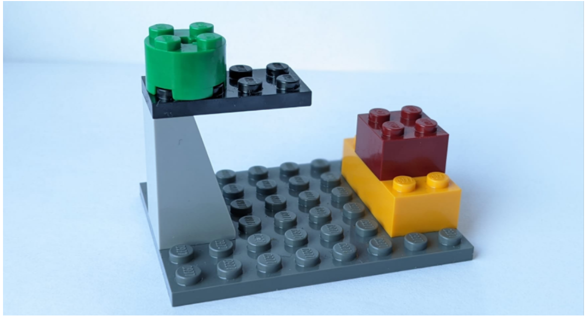

(a)

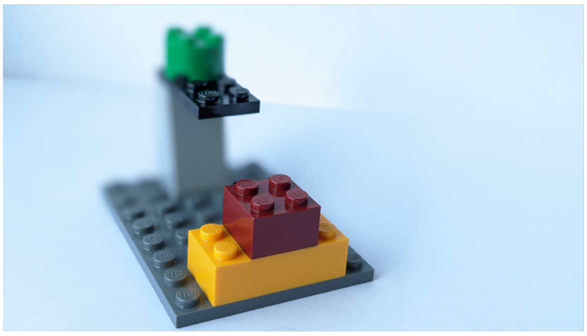

(b)

Figure 5: Prototype Model 4 administered in Trial 4.

2.6 Figure 6

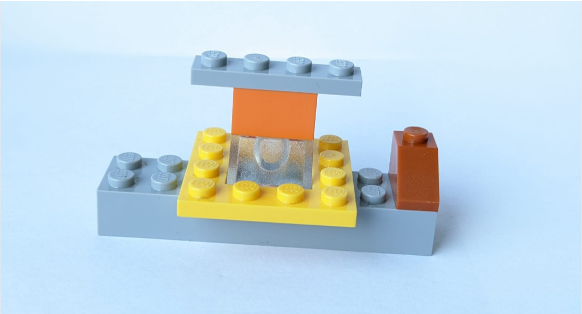

(a)

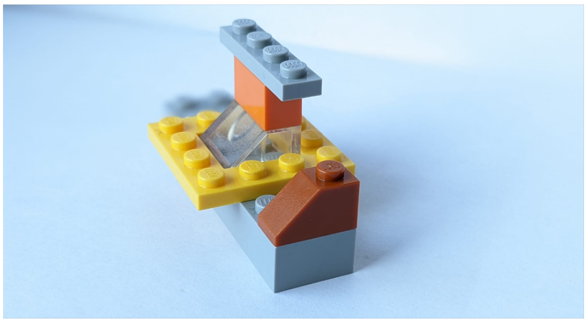

(b)

Figure 6: Prototype Model 5 administered in Trial 5.

2.7 Figure 7

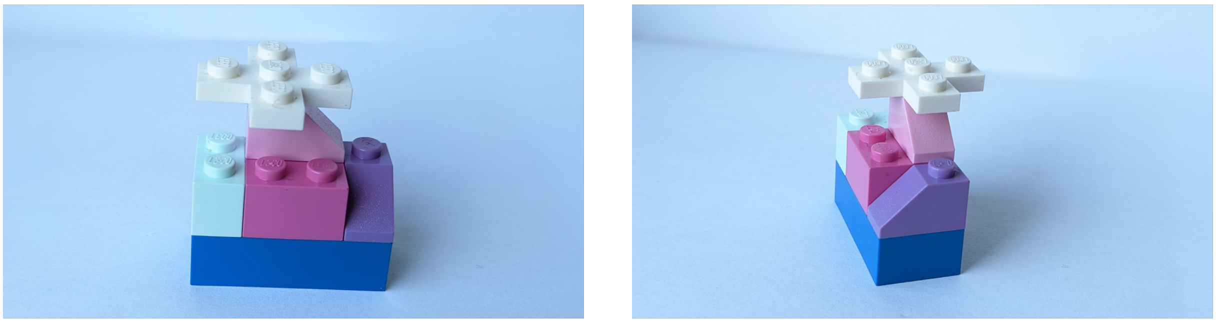

(a) (b)

Figure 7: Prototype Model 6 administered in Trial 6.

2.8 Figure 8

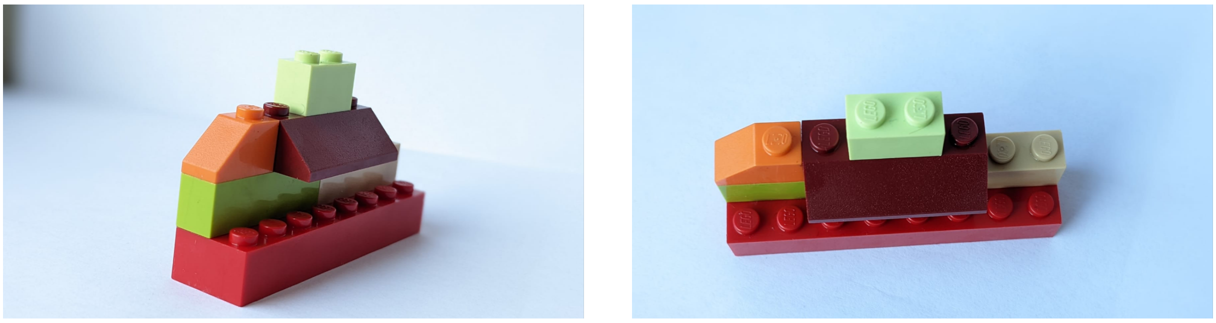

(a) (b)

Figure 8: Prototype Model 7 administered in Trial 7.

2.9 Figure 9

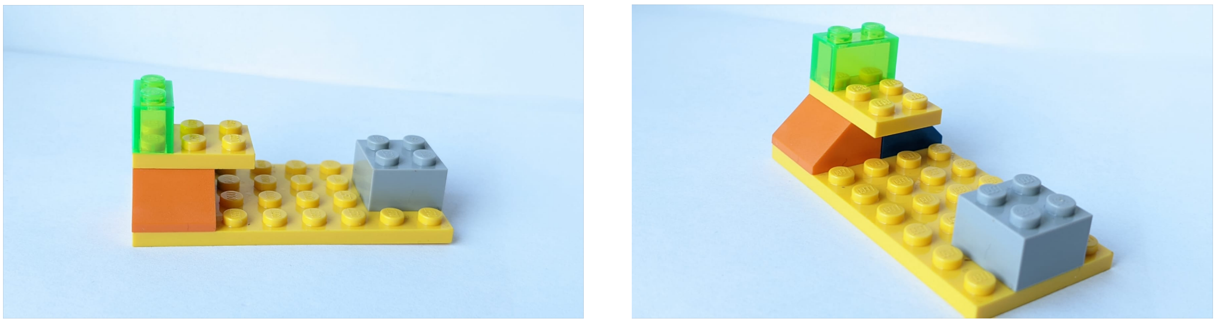

(a) (b)

Figure 9: Prototype Model 8 administered in Trial 8.

2.10 Figure 10

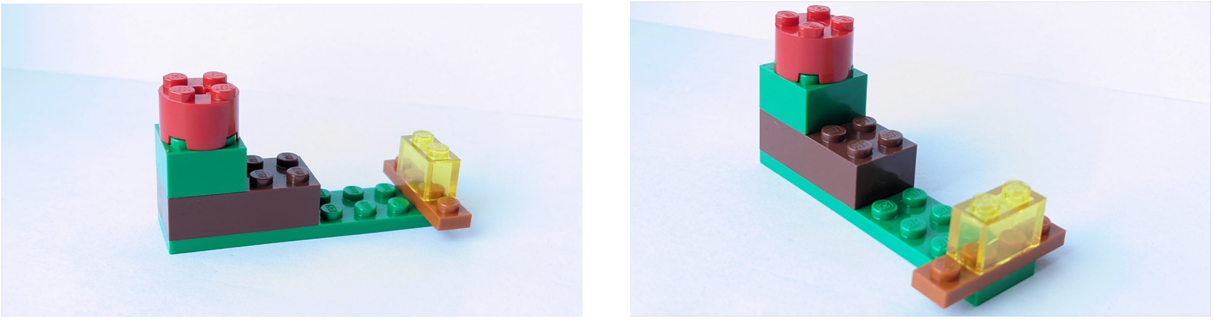

(a) (b)

Figure 10: Prototype Model 9 administered in Trial 9.

2.11 Figure 11

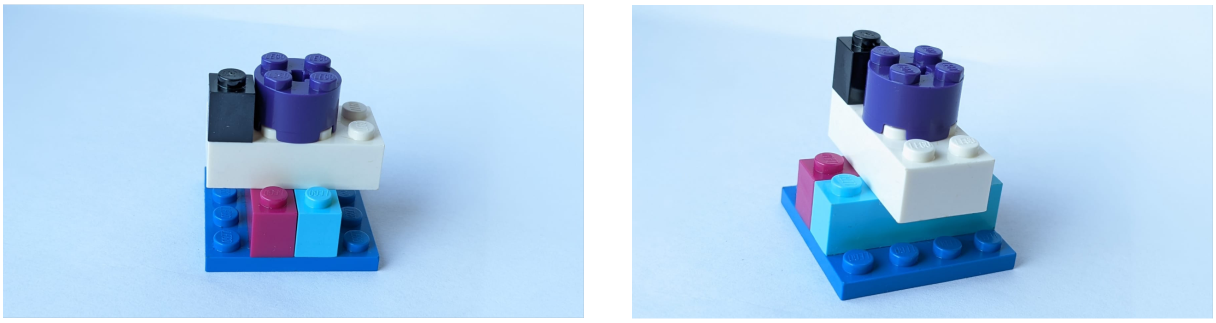

(a) (b)

Figure 11: Prototype Model 10 administered in Trial 10.

3 COVID-19 safety measures

Data collection in Vietnam took place while governmental COVID-19 safety regulations were in place. At the beginning of the data collection period, these measures affected half of our Vietnamese sample ($N = 18$ dyads or 45%). To ensure the safety of our participants, we implemented a set of additional measures. These measures affected the presence of the research stuff, the experimental setup, and the procedure.

3.1 Research team

Research team size was reduced to two people: the technician working at the study location and the experimenter attending via Zoom. **The technician** was required to wear a medical mask, a protective face shield, and medical gloves. The technician's responsibilities included:

1. setting up recording equipment and participants' working spaces;
2. disinfecting the study materials and working surfaces after each experimental session;

3. meeting participants outside of the study location (maintaining the distance of at least 2 meters) and explaining how to navigate to the study room.

The experimenter participated online via Zoom. The experimenter's responsibilities included:

1. administering consent forms and self-report questionnaires;
2. instructing participants;
3. supervising the change of trials and providing model feedback at the end of each trial;
4. monitoring participants for rule violations;
5. debriefing participants after the session;
6. supervising the collection of monetary rewards.

3.2 Changes in the experimental setup

We used the default recording setup with additional changes illustrated in Figure 12.

1. In addition to the transparent safety screen, we also installed a transparent PVC curtain to fully separate participants' working spaces.
2. In each working space, we placed a laptop facing each participant, hand sanitisers, new medical mask, consent forms, self-report questionnaires, pens, and clip-on microphones with transmitters.
3. Next to director's working space, we arranged bags with prototype models that were labelled according to trial numbers.
4. Next to builder's working space, we placed opaque cloth for separating participants in the hidden condition.

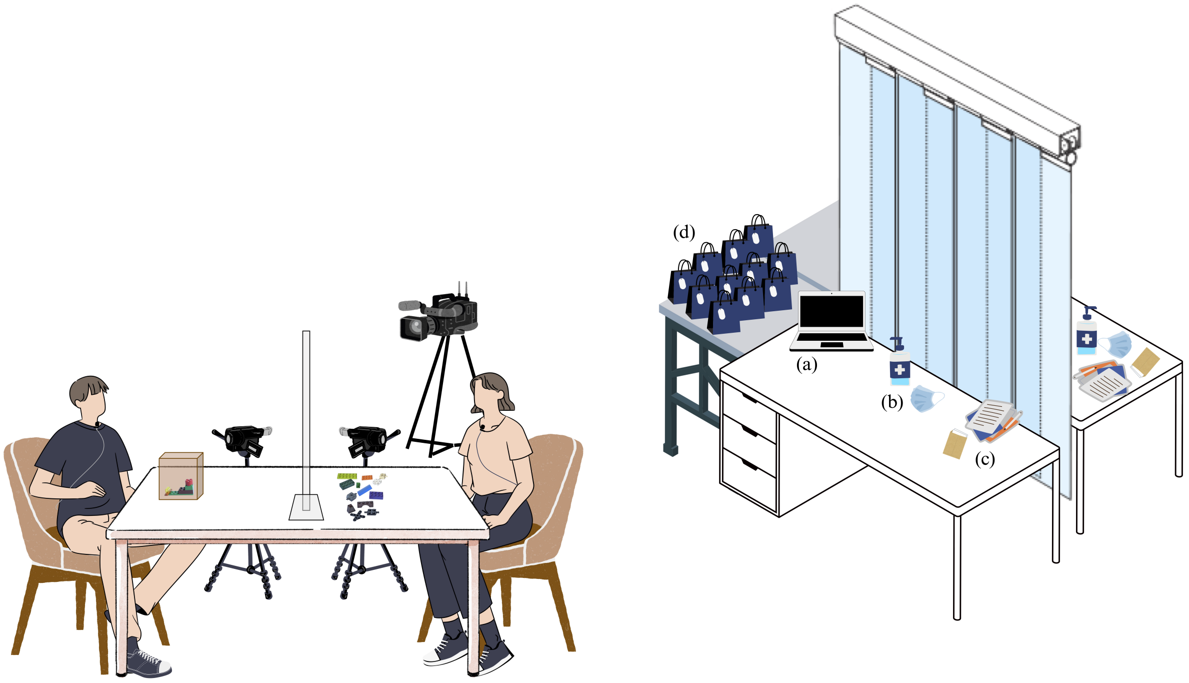

(a) Default setup

(b) Novel setup

Figure 12: **Default setup (left) compared to the novel setup (right).** Illustration of the director's working space in the novel setup: (a) a laptop, (b) hand sanitiser and a medical mask, (c) a consent form, a self-report questionnaire, a pen, and an envelop with monetary reward, (d) labelled bags with prototype models. The novel setup also included an opaque box for concealing prototype models, LEGO blocks, and recording equipment from the default setup.

3.3 Changes in the procedure

1. To ensure the accurate implementation of all COVID-safety measures, we allocated at least 1h 30mins of participation time per dyad (given the average length of an experimental session of 30-40 minutes).

2. Participants arrived separately, with an interval of approx. 5-10 minutes. Each of them followed the instructions provided by the technician and sat at the table with their initials on it.

3. On the table in front of them, participants found the laptop with an ongoing Zoom call. They were greeted by the experimenter via Zoom and asked to disinfect their hands and put on the new medical masks provided by the research team.

4. The experimenter administered consent forms and self-report questionnaires, then introduced the game rules and initiated the test model building.

5. The experimenter demonstrated how participants should clip lavalier microphones and transmitters to their clothes.

6. The experimenter asked each participant to turn off the laptop screen brightness completely, so that participants could not see each other through the web cameras during the experiment. Participants left their web cameras on, to allow the experimenter monitor them.
7. Directors were responsible for changing models between trials without exposing them to builders. Models were placed in labelled paper bags next to the director's working space.
8. After participants finished building each model, they showed their respective models to the experimenter via web cameras, and the experimenter explained any differences.
9. When it was time to change conditions, the experimenter instructed builders to place the cloth on the separation screen.
10. When the session was over, participants were asked to sign a receipt and collect their monetary rewards.
11. Participants disinfected their hands again and left the study location separately, with an interval of several minutes.
12. The experimenter informed the technician that it was safe to prepare the study location for the next dyad. The technician reset recording equipment, disinfected the room, including the experimental materials and table surfaces, then placed new medical masks and participant documents on the tables, and changed the cards containing participants' initials.
13. The technician left the room to wait for the next dyad outside.

4 Distribution of marker forms between stand-alone and within-turn contexts

Here, we provide the detailed distributions of each coordination marker form as used in *stand-alone contexts* (i.e. as turn-foregoing tokens produced in isolation) vs. *within-turn contexts* (i.e. as turn-prefacing or turn-final tokens), as partially reported in Sections 3.3.2 - 3.3.4 of the manuscript. Repetitions of the sane form (e.g. *ouais ouais ouais*) within the same turn were considered a single occurrence.

4.1 Swiss French

marker form	stand-alone (%)	within-turn (%)	total
ouais	1703 (58.4%)	1211 (41.6%)	2914 (100%)
OK	1398 (59.5%)	952 (40.5%)	2350 (100%)
mm-hm / mm	822 (96.8%)	27 (3.2%)	849 (100%)
oui	322 (64.3%)	179 (35.7%)	501 (100%)
voilà	258 (62.5%)	155 (37.5%)	413 (100%)
exactement / exact	124 (49.8%)	125 (50.2%)	249 (100%)
d'accord	56 (62.2%)	34 (37.8%)	90 (100%)
parfait	52 (63.4%)	30 (36.6%)	82 (100%)
top / tip top	25 (89.3%)	3 (10.7%)	28 (100%)
magnifique	14 (66.7%)	7 (33.3%)	21 (100%)
super	10 (71.4%)	4 (28.6%)	14 (100%)
tac	2 (100%)		2 (100%)
effectivement	1 (100%)		1 (100%)
mixed	458 (35.4%)	835 (64.6%)	1293 (100%)

Table 1: Occurrences of Swiss French marker forms and their proportional distributions between stand-alone vs. within-turn contexts.

4.2 Vietnamese

marker form	stand-alone (%)	within-turn (%)	total
rồi	1006 (54.7%)	834 (45.3%)	1840 (100%)
ô kê	995 (66.0%)	513 (34.0%)	1508 (100%)
ừm/ừm-hứm	951 (88.8%)	120 (11.2%)	1071 (100%)
ờ/ừ	478 (54.2%)	404 (45.8%)	882 (100%)
đúng/đúng rồi	251 (41.4%)	356 (58.6%)	607 (100%)
được/được rồi	116 (52.3%)	106 (47.7%)	222 (100%)
dạ/vâng/dạ vâng	87 (79.8%)	22 (20.2%)	109 (100%)
chuẩn	5 (18.5%)	22 (81.5%)	27 (100%)
chính xác	9 (64.3%)	5 (35.7%)	14 (100%)
mixed	406 (43.0%)	539 (57.0%)	945 (100%)

Table 2: Occurrences of Vietnamese marker forms and their proportional distributions between stand-alone vs. within-turn contexts.

4.3 Shipibo-Konibo

marker form	stand-alone (%)	within-turn (%)	total
aya/ya	3094 (54.9%)	2544 (45.1%)	5638 (100%)
jejen/ajan/jem/mm	1814 (68.1%)	849 (31.9%)	2663 (100%)
moa	250 (77.2%)	74 (22.8%)	324 (100%)
aita	83 (38.4%)	133 (61.6%)	216 (100%)
jaska-	69 (66.3%)	35 (33.7%)	104 (100%)
jakon	5 (16.1%)	26 (83.9%)	31 (100%)
listo	17 (77.3%)	5 (22.7%)	22 (100%)
okey	10 (100%)		10 (100%)
eri	4 (80.0%)	1 (20.0%)	5 (100%)
perfecto	1 (100%)		1 (100%)
exacto		1 (100%)	1 (100%)
mixed	392 (45.1%)	478 (54.9%)	870 (100%)

Table 3: Occurrences of Shipibo-Konibo marker forms and their proportional distributions between stand-alone vs. within-turn contexts.

4.4 Distribution of horizontal and vertical markers between stand-alone and within-turn contexts

turn position	transition	occurrence (%)
stand-alone	horizontal	2850 (55.3%)
	vertical	2304 (44.7%)
within-turn	horizontal	1770 (48.2%)
	vertical	1900 (51.8%)

Table 4: Distributions in Swiss French.

turn position	transition	occurrence (%)
stand-alone	horizontal	2023 (47.0%)
	vertical	2281 (53.0%)
within-turn	horizontal	1214 (41.6%)
	vertical	1707 (58.4%)

Table 5: Distributions in Vietnamese.

turn position	transition	occurrence (%)
stand-alone	horizontal	3062 (**53.4%**)
	vertical	2677 (**46.6%**)
within-turn	horizontal	2140 (**51.6%**)
	vertical	2006 (**48.4%**)

Table 6: Distributions in Shipibo-Konibo.

5 Distribution of marker forms between participant roles (directors vs. builders)

Here, we provide by-form counts of coordination markers as deployed by builders vs. directors.

5.1 Swiss French

marker	role		total
	builder	*director*	
ouais	2275 (**55.7%**)	1812 (**44.3%**)	4087 (**100%**)
OK	2057 (**69.9%**)	886 (**30.1%**)	2943 (**100%**)
mm-hm / mm	854 (**94.2%**)	53 (**5.8%**)	907 (**100%**)
repetitions	530 (**59.4%**)	363 (**40.6%**)	893 (**100%**)
oui	434 (**61.1%**)	276 (**38.9%**)	710 (**100%**)
voilà	44 (**6.6%**)	618 (**93.4%**)	662 (**100%**)
exactement / exact	25 (**4.6%**)	513 (**95.4%**)	538 (**100%**)
parfait	47 (**26.0%**)	134 (**74.0%**)	181 (**100%**)
d'accord	109 (**73.2%**)	40 (**26.8%**)	149 (**100%**)
other	52 (**42.3%**)	71 (**57.7%**)	123 (**100%**)

Table 7: By-form distribution of marker counts between participant roles in Swiss French.

5.2 Vietnamese

marker	role		total
	builder	*director*	
rồi	1831 (**70.9%**)	753 (**29.1%**)	2584 (**100%**)
ô kê	1333 (**66.9%**)	659 (**33.1%**)	1992 (**100%**)
ừm/ừm-hứm	943 (**77.8%**)	269 (**22.2%**)	1212 (**100%**)
ờ/ừ	553 (**46.5%**)	636 (**53.5%**)	1189 (**100%**)
repetitions	963 (**83.4%**)	191 (**16.6%**)	1154 (**100%**)
đúng/đúng rồi	78 (**8.5%**)	837 (**91.5%**)	915 (**100%**)
được/được rồi	75 (**26.2%**)	211 (**73.8%**)	286 (**100%**)
dạ/vâng/dạ vâng	129 (**75.0%**)	43 (**25.0%**)	172 (**100%**)
chuẩn	5 (**9.4%**)	48 (**90.6%**)	53 (**100%**)
chính xác	3 (**6.7%**)	42 (**93.3%**)	45 (**100%**)

Table 8: By-form distribution of marker counts between participant roles in Vietnamese.

5.3 Shipibo-Konibo

marker	role		total
	builder	*director*	
aya/ya	3537 (**54.8%**)	2920 (**45.2%**)	6457 (**100%**)
jejen/ajan/jem/mm	1145 (**35.1%**)	2119 (**64.9%**)	3264 (**100%**)
repetitions	465 (**60.6%**)	302 (**39.4%**)	767 (**100%**)
moa	220 (**43.9%**)	281 (**56.1%**)	501 (**100%**)
jaska-	52 (**14.3%**)	312 (**85.7%**)	364 (**100%**)
aita	4 (**2.1%**)	187 (**97.9%**)	191 (**100%**)
other	64 (**34.8%**)	120 (**65.2%**)	184 (**100%**)

Table 9: By-form distribution of marker counts between participant roles in Shipibo-Konibo.
